# On-Surface Synthesis of Nonbenzenoid PAHs Using Intermolecular π‑Radical C–C Coupling

**DOI:** 10.1021/jacs.5c12864

**Published:** 2025-11-19

**Authors:** Federico Frezza, Erik Misselwitz, Qifan Chen, Pingo Mutombo, Frank Rominger, Ana Sánchez-Grande, Milan Kivala, Pavel Jelínek

**Affiliations:** † 86889 Institute of Physics of the Czech Academy of Sciences, Cukrovarnická 10, 16200 Prague, Czech Republic; ‡ Organisch-Chemisches Institut, Universität Heidelberg, Im Neuenheimer Feld 270, 69120 Heidelberg, Germany; § CATRIN - RCPTM, Palacký University Olomouc, Šlechtitelů 27, 77146 Olomouc, Czech Republic

## Abstract

On-surface synthesis has emerged as a new research field, ideal for designing low-dimensional carbon-based nanomaterials. One of the central problems with this synthetic approach is the understanding of reaction mechanisms, which is a key point for advancing the design of novel, highly selective reactions. The concept of π-radical-mediated reactions has been rarely considered in the context of on-surface synthesis so far. Here, we demonstrate that a π-radical-mediated reaction can provide an efficient mechanism of regioselective carbon–carbon coupling. Namely, π-radical coupling enables the dimerization of two π-expanded acenaphthene units, which facilitates the formation of complex nonbenzenoid PAHs. Our work contributes to the understanding of reaction mechanisms at the fundamental level, thus bridging the gap between in-solution radical chemistry and on-surface synthesis. We demonstrate a highly selective reaction in which the crucial C–C coupling step proceeds without direct catalytic involvement of the gold surface. This mechanistic insight suggests that π-radical coupling is a promising strategy that could be potentially expanded to inert surfaces, providing suitable π-radical activation.

## Graphical abstract

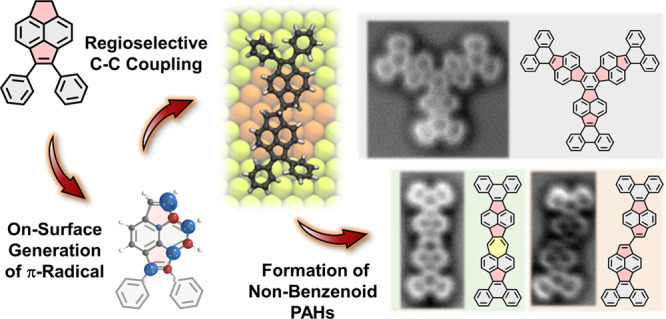

## Introduction

Over the last two decades, a new organic synthetic approach on metallic surfaces under ultrahigh vacuum (UHV) conditions has been explored, known as on-surface synthesis (OSS).^1–3^ OSS has demonstrated its potential to synthesize novel chemical compounds hardly available by traditional synthetic approaches, including atomically precise graphene nanoribbons^4–7^ and polymers,^8,9^ presenting nontrivial electronic structures.^10–13^ Particularly interesting is the possibility of synthesizing open-shell polyaromatic hydrocarbons (PAHs) presenting π-radicals, including systems with high-spin ground states, on gold surfaces.^14–19^ Unlike σ-radicals, where the unpaired electron is located in a strongly localized σ orbital, π-radicals have unpaired electrons delocalized in π orbitals (see Figure S1, σ and π used as symmetry descriptors according to the definition in the IUPAC Gold Book^20^). Additionally, π-radicals are often stabilized by delocalization, as illustrated in Figure 1b, making them less reactive than σ-radicals, which lack delocalization as a stabilizing factor (exemplified in Figure 1 for a π-expanded acenaphthene unit). Thus, the inert UHV environment and the 2D confinement effect imposed by the noble gold surface create ideal conditions to stabilize π-radicals. Moreover, the chemical and electronic structures of π-radicals can be examined in situ with unprecedented spatial resolution using UHV low-temperature scanning probe microscopy (SPM).^15,21^ Nevertheless, new reaction mechanisms need to be explored to expand the potential of the OSS approach.

**Figure 1:**
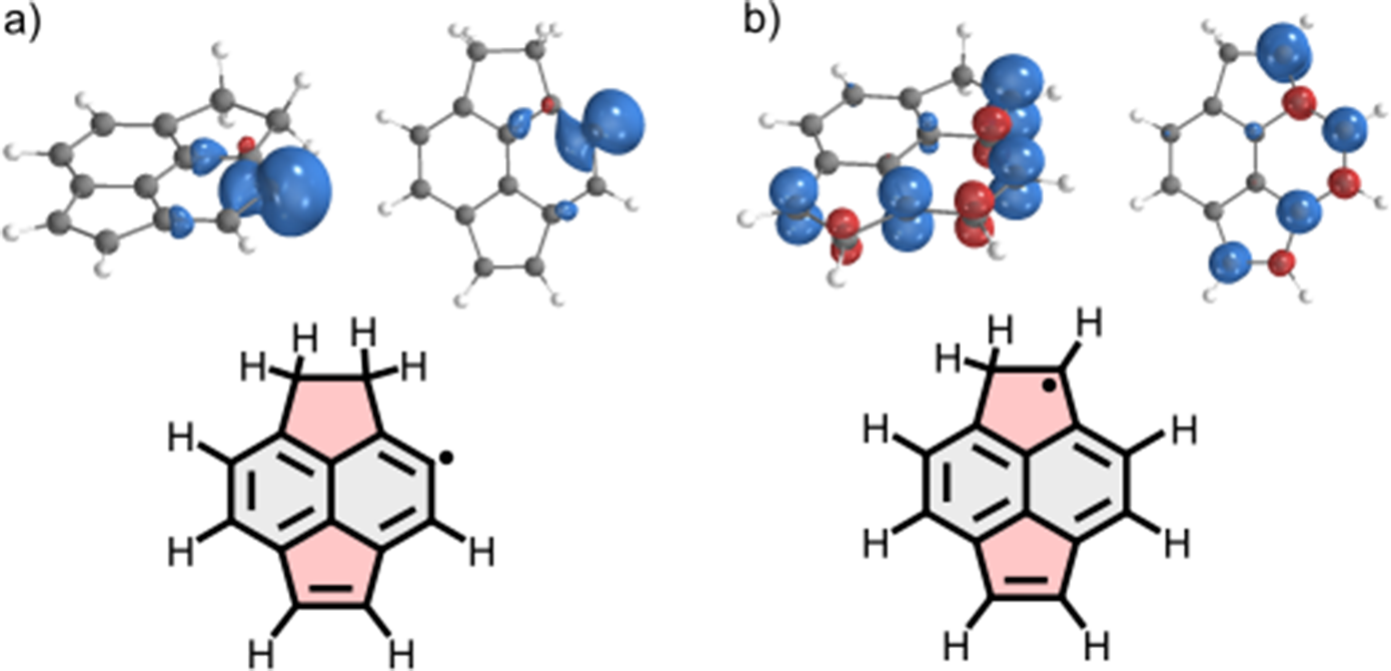
Spin density plots of a π-expanded acenaphthene unit presenting (a) σ-radical versus (b) π-radical, showing distinct spin delocalizations.

The great success of OSS is often attributed to the catalytically active role of the metal surface, which opens new reaction pathways, as demonstrated in numerous works.^22,23^ Besides all works where the metal substrate was crucial for inducing new on-surface reactions, the “catalytic role of the metal substrate” remains an ambiguous concept. Moreover, more in-depth insight into the reaction mechanisms available in UHV OSS is still missing. To rationalize the difference between in-solution and UHV OSS, it is helpful to compare established concepts of reaction mechanisms from both synthetic approaches. In solution, most reactions exhibit a heterolytic character, forming ionic intermediates that are often stabilized by polar solvents. However, the situation on surfaces under UHV conditions is quite different. In this context, it is essential to note that the formation of ionic intermediates on metal surfaces under UHV conditions is unlikely: the metal surface acts as a reservoir of infinite electrons/holes, which typically causes fast quenching of ionic species at metal surfaces. This fast charge transfer substantially reduces the significance of heterolytic reactions on metal surfaces. Thus, in principle, homolytic reactions with radical intermediates should play a much more critical role in on-surface UHV chemistry than in-solution chemistry.

So far, most on-surface UHV reactions have been based on Ullmann coupling,^24^ initiated by the homolytic cleavage of the C–X bond (X = halogen), assisted by the metal surface.^23^ Here, the cleavage mechanism of the σ-bond creates a highly reactive σ-radical species, which tends to be passivated, forming organometallic intermediates.^25–27^ Indeed, theoretical investigations of the on-surface reaction mechanism of C–X bond cleavage indicate that radical species are passivated by direct coordination to a surface atom^23,28^ or a single adatom.^29^ Recently, the catalytic role of single adatoms in activating C–H^30–32^ and C–C bonds^33^ was theoretically recognized. Here, the single gold adatom not only reduces the activation barrier but also quenches the radical character of intermediates by the formation of an organometallic complex, substantially lowering their energy and opening new reaction pathways. However, forming a strong dative-covalent bond between a carbon-centered σ-radical and a metal adatom can have undesirable effects on the resulting chemical products. In many cases, it has been observed that the high stability of organometallic intermediates hinders the formation of the desired C–C bonds.^34,35^

From this perspective, it is not surprising that the concept of radical chemistry^36,37^ has been rarely discussed in the context of OSS.^38–40^ Recently, the intermolecular radical transfer reaction from a dehalogenated aryl halide, forming a surface-stabilized phenyl σ-radical, to a terminal alkyne on metal surfaces has been demonstrated,^41^ highlighting the relevance of radical chemistry in the OSS framework. Recent studies highlighted the potential of such pathways, demonstrating C–C coupling reactions involving radical intermediates.^42,43^ While these works showcase the synthetic utility of these reactions, a fundamental investigation into the mechanism of π-radical dimerization and the precise role of the underlying surface in the coupling step is still needed to generalize this approach. In this regard, the commonly accepted idea that unpaired electrons of radicals interact strongly with the metal substrate or adatoms contrasts with the recent progress in the OSS of carbon-based π-magnets on metal surfaces, as mentioned above. This dichotomy can be rationalized by the different reactivities of unpaired electrons, depending on the σ or π orbital character that hosts them. In the case of σ-radicals, where unpaired electrons reside in σ molecular orbitals, they tend to interact strongly with metallic substrates, forming surface-passivated radicals. On the contrary, in π-radicals, unpaired electrons are primarily located in π molecular orbitals, and their radical character is, at least partially, retained on gold surfaces. From this perspective, π-radicals appear to be good candidates for a radical coupling mechanism, once they are properly activated on surfaces.

Here, we report the OSS of nonbenzenoid PAHs employing π-radical coupling. We demonstrate that a π-expanded acenaphthene (1,2-dihydroacenaphthylene) unit can be employed for the regioselective formation of a π-radical on the 5-membered ring after C–H bond cleavage assisted by a single Au adatom. The formation of the π-radical facilitates selective intermolecular radical-mediated C–C coupling, leading to the formation of dimers. In the second reaction step, further annealing at 400 °C facilitates the formation of three different nonbenzenoid PAHs, enabled by single gold adatom catalysis (see Figure 2). We employed high-resolution SPM techniques to characterize the chemical and electronic structures of the three products. The reaction steps are rationalized by free-energy QM/MM simulations, revealing that while the Au(111) surface serves multiple crucial roles throughout the mechanism by supplying metal adatoms and facilitating dehydrogenation steps, the key radical-mediated dimerization step itself occurs with the surface acting only as a 2D support. Our findings show the potential of π-radical reactions in UHV OSS. For our chosen precursor, the initial π-radical is generated via C–H bond cleavage assisted by a single Au adatom. However, the subsequent and highly selective intermolecular C–C coupling proceeds without further catalytic involvement from the surface. This mechanistic difference between radical formation and radical coupling is a key insight. It suggests that this synthetic approach could be expanded to semiconducting and insulating substrates in the future, provided that π-radicals are activated by noncatalytic means such as light or tip manipulation. This π-radical coupling mechanism can be considered as an alternative scenario for the formation of decacyclene derivatives, previously synthesized either in solution by aldol cyclotrimerization^44^ or by strain-induced ring contraction.^45^

**Figure 2:**
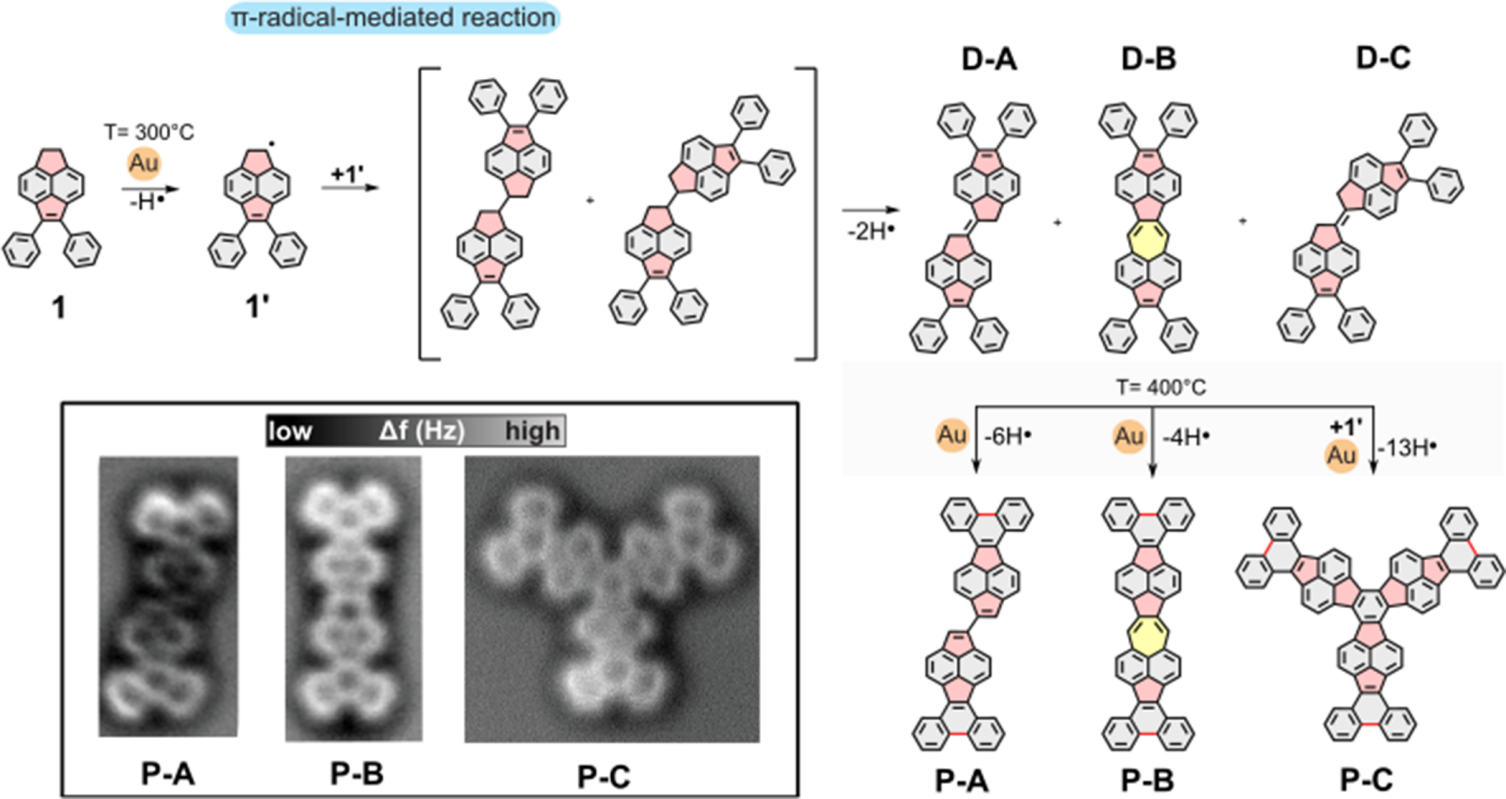
Synthetic route toward the on-surface synthesis of nonbenzenoid PAHs via π-radical coupling. The inset shows the experimental nc-AFM images of the three products.

## Results and Discussion

The synthesis of the acenaphthene precursor (**1**) has been performed following the synthetic protocol described in Scheme S1 in the Supporting Information. The deposition of compound **1** on a Au(111) surface kept at room temperature (RT) leads to the formation of tetramers of **1**, following the herringbone reconstruction of the surface (see Figure S2). The molecular precursor was designed to afford the formation of a π-radical after the homolytic dehydrogenation of one of the C­(sp^3^) atoms on the acenaphthene unit. Thus, annealing at 300 °C induces the dimerization of **1** (see the scheme in Figure 2), forming chain-like structures coexisting with some intact molecules, where adjacent dimers interact with each other through the phenyl units, as shown in the scanning tunneling microscopy (STM) overview in Figure 3a. To elucidate the chemical structure of the dimers, we performed non-contact atomic force microscopy (nc-AFM) measurements employing a CO-functionalized tip. It should be noted that these molecules are not planar, so we can access limited information about the chemical structure. Nevertheless, we can resolve the structure of the “bridge” connecting the dimers. Interestingly, we find three distinct dimers, illustrated in Figure 3. Figure 3b shows the nc-AFM image of dimer A (**D-A**) and its corresponding chemical model, revealing the formation of a fulvalene-like moiety. Dimer C (**D-C**) also presents the equivalent connection corresponding to the cis configuration (Figure 3d). We observe a clear preference toward the formation of **D-A** with respect to **D-C**. We tentatively associate this effect with the template effect of the herringbone reconstruction together with possible steric interactions between the methylene hydrogens in **D-C**. Additionally, we observe the formation of a third dimer (**D-B**), illustrated in Figure 3c, containing one azulene unit (fused 5–7-membered rings).

**Figure 3:**
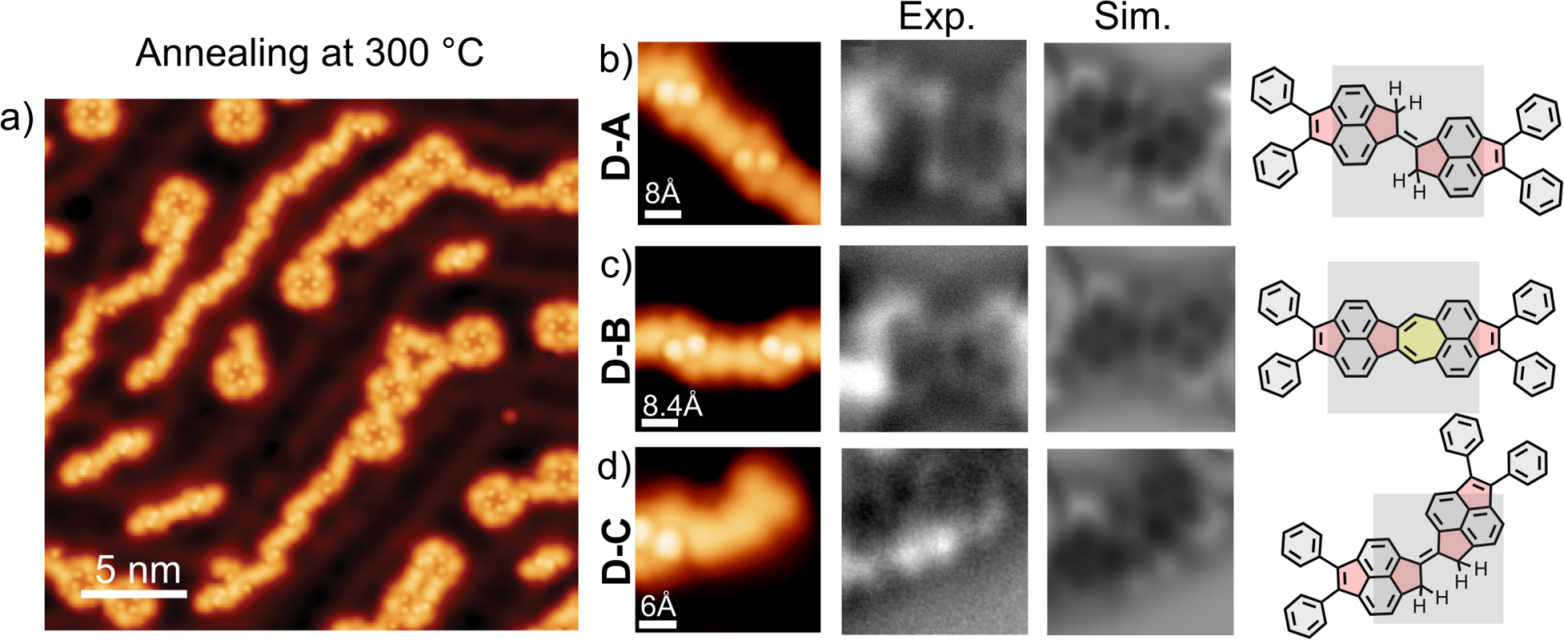
On-surface dimerization of precursor **1** after thermal activation at 300 °C. (a) STM overview image of the different dimers coexisting with unreacted precursors (Vb = 100 mV and It= 25 pA). (b) Constant-current STM image, experimental and simulated constant-height nc-AFM image, and chemical model of **D-A** (STM image: Vb= 80 mV and It = 10 pA. Nc-AFM image: Vb= 1 mV). (c) Constant-current STM image, experimental and simulated constant-height nc-AFM image, and chemical model of **D-B** (STM image: Vb= 90 mV and It = 10 pA. Nc-AFM image: Vb= 1 mV). (d) Constant-current STM image, experimental and simulated constant-height nc-AFM image, and chemical model of **D-C** (STM image: Vb= 50 mV and It = 10 pA. Nc-AFM image: Vb= 1 mV). Gray squares superimposed on chemical models indicate the part of the molecule represented in the corresponding nc-AFM image.

To understand the mechanism of the on-surface reaction mediated by π-radicals, we performed QM/MM calculations. In the first step, we assume that the presence of diffusing single Au adatoms facilitates the homolytic cleavage of the C­(sp^3^)–H bond. Our free-energy QM/MM calculations predict a relatively low activation barrier for the dehydrogenation step of 0.67 eV, accessible under the reaction conditions (see Figure 4a,b). This homolytic cleavage generates π-radical acenaphthene intermediates, which are crucial in understanding the mechanistic aspects of the reaction. In contrast to σ-radical reaction intermediates, our calculations show a relatively weak interaction of the gold adatom with the π-radical. This effect is caused by the presence of a planar C–H bond, which hinders direct interaction of the gold adatom with the radical carbon atom. This effect also facilitates C–C coupling between two π-radical acenaphthene units, which does not require the assistance of a Au adatom or the surface. According to our QM/MM calculations, π-radical-mediated coupling is the most favorable pathway, with a barrier of ≈1 eV (see Figure 4a,c), resulting in the formation of thermodynamic stable dimers with an energy yield of 20 kcal/mol (0.87 eV). It should be noted that the energy barrier is substantially lower with respect to further dehydrogenation of the acenaphthene unit (see Figure S3), which would lead to the formation of an additional double bond in **1’**.

**Figure 4:**
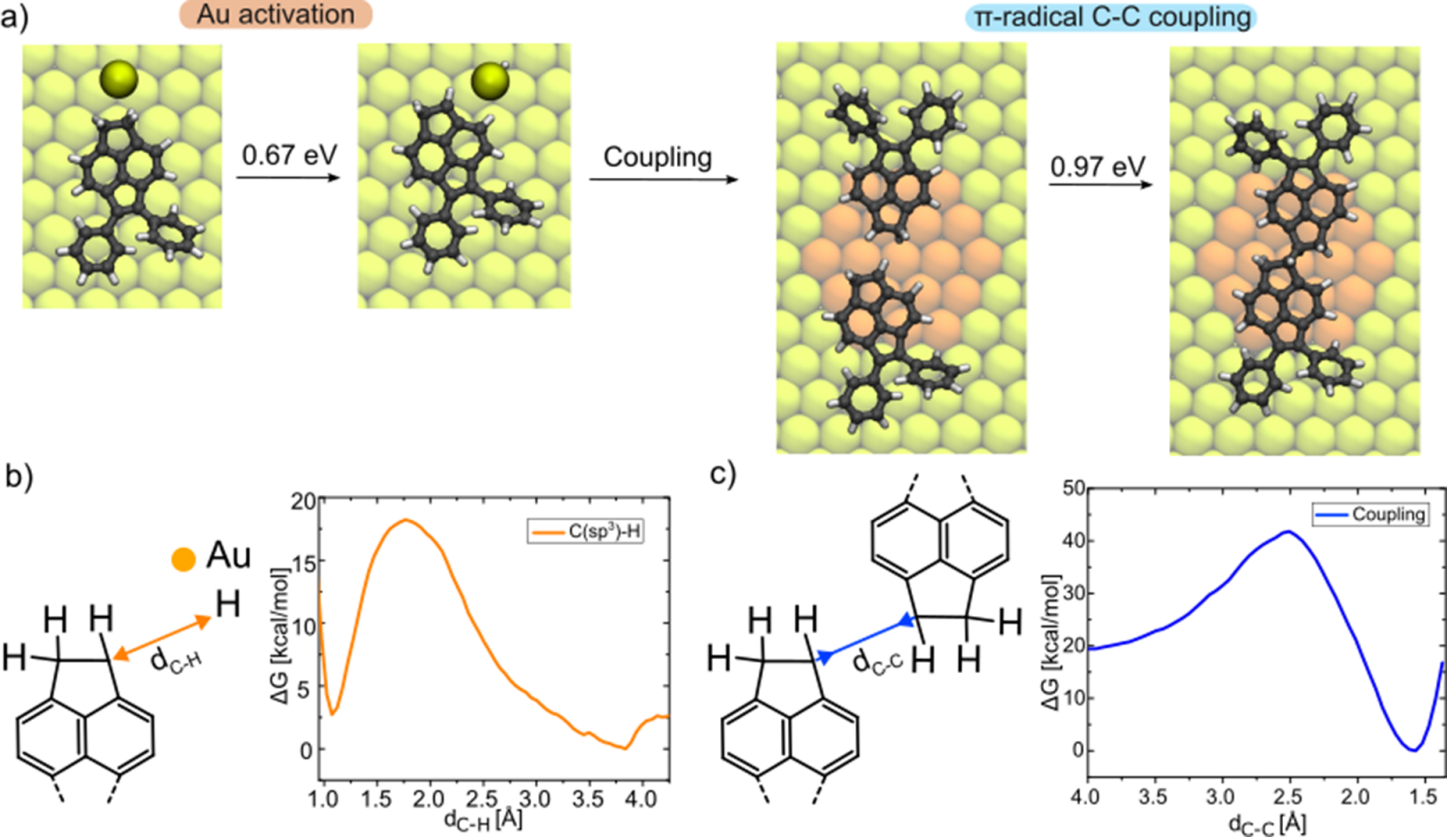
QM/MM free-energy calculations of π-coupling dimerization. (a) Calculations of Au-assisted C­(sp^3^)H cleavage and π-radical-mediated C–C coupling. C, H, Au adatoms, Au surface atoms in the MM region, and Au surface atoms in the QM region are represented in black, white, golden yellow, light yellow, and orange balls, respectively. (b,c) Free-energy profiles of Au-assisted dehydrogenation and π-radical coupling, respectively.

We can rationalize this reactivity due to the “stability” of the free π-radical **1’** on the Au(111) surface. Carbon-centered radicals can get stabilized by different strategies, such as imposing steric protection or a delocalized spin density distribution. In general, the possibility of stabilizing highly polyradical π-radical PAH species on Au(111) is attributed to strong delocalization of unpaired electrons in the π-system.^17,46,47^ Figure 1 shows a comparison of the spin density of the π-radical generated after the initial Au activation step and the hypothetical σ-radical generated. It should be noted that for simplicity, an acenaphthene unit was employed without the two lateral phenyl substituents. Here, the π-conjugated core of the molecule clearly contributes to stabilizing the π-radical, together with the steric protection of π-radicals by the adjacent −CH_2_– group from the Au surface. Moreover, the π-radical is sterically protected by the in-plane hydrogen bonded to the carbon radical, denying Au adatoms access to the radical. All these factors contribute to the highly selective dimerization of the π-radical acenaphthylene intermediates, with relatively low barriers of ≈1 eV. Despite the extended delocalization of the π-radical shown in Figure 1, we observe a highly selective reaction, corroborated by our simulations that predict a much bigger activation barrier of ∼1.5 eV for C–C coupling between a π-radical and aromatic carbons (see Figure S4). An even larger reaction barrier of 3.56 eV is predicted for coupling between an activated molecule **1’** and an intact precursor (Figure S4). This explains the large regioselectivity of the π-radical-mediated C–C coupling between the acenaphthene units.

After the C–C coupling reaction, we observe a bifurcation of the reaction toward the formation of **D-A**, **D-B**, and **D-C**. The radical intermediate becomes asymmetric on the surface, resulting in two possible connections, as shown in Figure 2. These two routes were investigated by QM/MM calculations to rationalize the pathways involved in the formation of the experimentally observed compounds. The first route toward the synthesis of **D-A** is shown in Figure S5, where the active role of Au adatoms is evident, significantly reducing the energy barriers (≤1.00 eV). As a comparison, the first dehydrogenation step without the assistance of a Au adatom presents a significantly higher energy barrier of 1.73 eV. The second route is shown in Figure S6. Our proposed mechanism illustrates the energetically most favorable pathways toward the formation of **D-B** and **D-C**. Our calculations predict that the formation of the azulene unit involves the formation of a cyclobutene intermediate, as described in a previous work where the reaction is induced by light and in vacuo.^48^ Altogether, the calculations highlight the role of both Au adatoms and the surface.

Further annealing of the sample at 400 °C triggers a cyclodehydrogenation reaction between phenyls, inducing the complete planarization of the molecules and resulting in the synthesis of different planar nonbenzenoid PAHs (see Figure 5a). The planarity of the molecules allows unambiguous elucidation of the chemical structure by high-resolution nc-AFM measurements with a CO-tip. We identified three main products, named **P-A**, **P-B**, and **P-C** (chemical models and nc-AFM images are shown in the bottom panel of Figure 2). The formation of **P-A** and **P-B** is achieved after the cyclodehydrogenation reaction of the phenyl “legs” of **D-A** and **D-B** toward the generation of phenanthrene units (the formation of **P-A** involves an additional dehydrogenation step of the fulvalene-based bridge). Details about a possible cyclodehydrogenation reaction mechanism obtained by employing QM/MM calculations are provided in Figure S7. The **P-C** product consists of a decacyclene-derived trimer. Considering the relatively large size of the product and the complexity of the possible reaction pathways, the significant computational cost limits the information we can provide about the reaction mechanism, leading to the formation of **P-C**. Nevertheless, based on the experimental observation that unreacted molecules coexist with the dimers after annealing at 300 °C (see Figure S8), we propose that **D-C** undergoes another coupling reaction with unreacted monomers to form **P-C**. A statistical analysis of over 700 molecules reveals that the reaction is highly selective toward the three main products **P-A** (∼77%), **P-B** (∼8%), and **P-C** (∼6%). The remaining minority species (∼9%) result from less favorable side reactions, such as the attack of a π-radical onto an aromatic carbon to form a T-shaped trimer (Figure S9). The lower yield of such products is in agreement with the QM/MM simulations, predicting a significantly higher activation barrier for this reaction (Figure S4).

**Figure 5:**
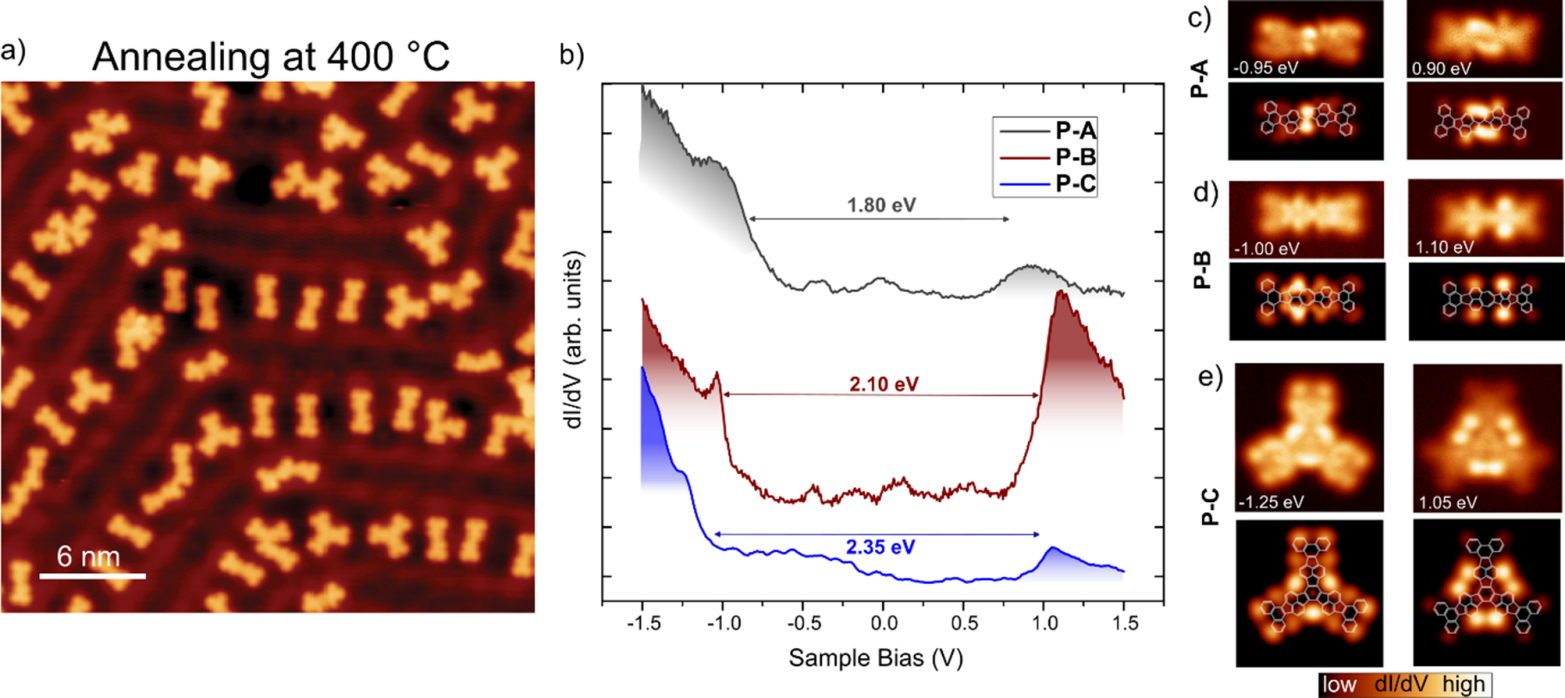
Electronic properties of nonbenzenoid PAHs. (a) STM overview image after annealing at 400 °C toward the synthesis of **P-A**, **P-B**, and **P-C** (Vb= −100 mV and It= 35 pA). (b) Differential conductance d*I*/d*V* spectra showing the frontier resonances of **P-A**, **P-B**, and **P-C**. (d–f) Experimental d*I*/d*V* maps at the energies of the frontier resonances and simulated d*I*/d*V* maps of the HOMO and LUMO orbitals of **P-A**, **P-B**, and **P-C**, respectively.

Finally, we address the electronic properties of the three nonbenzenoid PAHs by scanning tunneling spectroscopy (STS) measurements. We performed a systematic study of the electronic properties by acquiring d*I*/d*V* curves over the PAHs to identify the frontier resonances (see Figure 5b), which were assigned to the negative ionic resonance (NIR) and positive ionic resonance (PIR) by comparing the experimental d*I*/d*V* maps at the energies of the resonances with simulated d*I*/d*V* maps of the HOMO and LUMO orbitals (Figure 5c). We observe an energy gap of 1.8 eV for **P-A**, slightly narrower than that of **P-B** that shows a gap of 2.1 eV. **P-C** shows a HOMO–LUMO gap of 2.35 eV, which is the largest of the three PAHs. These results are in qualitative agreement with HOMO–LUMO gaps calculated by DFT at the B3LYP­(D3BJ)/6-311G­(d,p) level of theory in the gas phase (Figure S10). Based on these calculations, the aromaticity in these compounds was assessed using Harmonic Oscillator Model of Aromaticity (HOMA) values (Figure S11), Nucleus Independent Chemical Shift (NICS) values (Tables S1–S3), and Anisotropy of the Induced Current Density (ACID) plots (Figure S12).^49–51^

For **P-A**, all indicators point to pronounced antiaromaticity in the 5-membered rings in both pyracylene (cyclopenta­[*fg*]­acenaphthylene) units. This is consistent with its lowest HOMO–LUMO gap among the series, in line with the known trend of decreasing HOMO–LUMO gaps in PAHs exhibiting antiaromatic character.^52,53^ In azulene-embedded **P-B**, antiaromatic character is observed in the 5-membered rings of the pyracylene subunit, while the 7-membered ring and the 5-membered ring in the acepleiadylene (cyclohept­[*fg*]­acenaphthylene) subunit appear essentially nonaromatic. In cyclotrimer **P-C**, all 5-membered rings, as well as the central 6-membered ring, exhibit modest antiaromaticity. Compared to **P-A**, the antiaromaticity in the 5-membered rings in the pyracylene subunits is significantly reduced. This reduced antiaromaticity in the scaffold results in the largest HOMO–LUMO gap for **P-C**, despite its apparently largest π system. Notably, a series of cyclotrimers featuring the same polycyclic scaffold as **P-C** has been synthesized in solution, which features exceptional redox properties in electrochemical studies.^54^ The structural data and aromaticity analysis for the series of compounds are consistent with the findings reported herein. These results highlight that the fusion pattern of nonbenzenoid rings plays an important role in governing their electronic properties and aromaticity, providing valuable insights for the design of materials with antiaromatic character.^55^ Additionally, the synthetic approach presented herein gives access to the preparation of structurally diverse cyclotrimers from simple building blocks, which could serve as precursors that, upon epitaxial elongation, yield single-chirality carbon nanotubes.^56,57^

## Conclusions

We have demonstrated the feasibility of π-radical chemistry in the context of UHV on-surface synthesis, establishing an alternative to the most employed OSS reactions. π-radical coupling, where the π-radicals retain their radical character due to weaker interactions with the substrate, enables selective intermolecular reactions. Specifically, we reported a highly selective C–C coupling reaction mediated by π-radicals. Importantly, the surface does not play an active chemical role in the C–C coupling step, where its contribution is limited to providing 2D support for the molecular reactants. This fundamental difference from traditional OSS reactions, such as Ullmann coupling, opens new mechanistic pathways where the substrate’s electronic properties are less critical.

While the initial generation of radicals in our study relies on the catalytically active Au(111) surface, the subsequent inert role of the substrate in the crucial π-radical coupling step is a key finding that opens promising avenues for future work, where designing molecular precursors that can be activated on semiconducting or insulating surfaces could allow for highly selective C–C coupling reactions without the need for a metallic catalyst.

## Supplementary Material



## Notes

Additional experimental and theoretical details, materials, methods, X-ray crystallographic data, QM/MM calculations, STM overviews, HOMA values, NICS values, and ACID plots (PDF)

## ∥

Organic Chemistry Department, Faculty of Chemical Sciences, Universidad Complutense Madrid, Pl. de las Ciencias 2, 28040 Madrid, Spain
