# Supplementary material for: On-Surface Synthesis of Nonbenzenoid PAHs Using Intermolecular π‑Radical C–C Coupling
Source: J Am Chem Soc. 2025 Nov 19;147(48):44123–30. doi: 10.1021/jacs.5c12864 (PMC12679639; doi:10.1021/jacs.5c12864)
Supplement: Supplementary file 1 [file ja5c12864_si_001.pdf]

Supporting Information

On-surface synthesis of non-benzenoid PAHs using intermolecular π-radical C-C coupling

Federico Frezza‡,[1] Erik Misselwitz‡,[2] Qifan Chen‡,[1] Pingo Mutombo,[1] Frank Rominger,[2] Ana Sánchez-Grande,[1,†] Milan Kivala*,[2] Pavel Jelínek*.[1,3]

1. Institute of Physics of the Czech Academy of Sciences, Cukrovarnická 10,16200 Prague, Czech Republic.

2. Organisch-Chemisches Institut, Universität Heidelberg, Im Neuenheimer Feld 270, 69120 Heidelberg, Germany

3. CATRIN - RCPTM, Palacký University Olomouc Šlechtitelů 27, 77146 Olomouc, Czech Republic

Contents

Methods 2
Synthesis 5
X-Ray Crystallographic Data 10
Figure S1. SOMOs of σ- and π-radicals 12
Figure S2. Self-assembly of precursor **1** on Au(111) 12
Figure S3. QM/MM calculations of possible reaction pathways after C-H cleavage 13
Figure S4. QM/MM calculations of possible C-C couplings 14
Figure S5. Proposed route towards the synthesis of **D-A** 15
Figure S6. Proposed route towards the synthesis of **D-B** and **D-C** 16
Figure S7. QM/MM calculations of cyclodehydrogenation reaction 17
Figure S8. STM overviews after RT deposition and annealing at 300 °C 17
Figure S9. STM overviews after RT annealing at 400 °C 18
Figure S10. Electronic structure based on DFT calculations in the gas phase 18
Figure S11. HOMA values 19
Table S1,2,3. NICS values 20
Figure S12. ACID plots 21
References 22

Methods

General

All solvents and reagents were purchased at reagent grade from commercial suppliers (Merck/Sigma-Aldrich, TCI, Thermo Fisher Scientific, Acros Organics, Honeywell, BLD Pharmatech) and used without additional purification. All reactions involving oxygen- or moisture-sensitive compounds were performed in heat gun dried glassware under an atmosphere of nitrogen using standard *Schlenk* techniques. The addition of oxygen- and moisture-sensitive solvents and reagents was carried out using nitrogen-flushed stainless-steel cannulas and plastic syringes. Thin layer chromatography was monitored on ALUGRAM aluminum plates from Macherey-Nagel, coated with 0.20 mm SiO_2, by irradiation with UV-light (λ= 365 and 254 nm). Flash column chromatography was carried out with SiO_2 from Macherey-Nagel (technical grade 60 M, pore size 60 Å, 40−63 µm particle size).

Nuclear Magnetic Resonance. Spectra were recorded at room temperature (295 K) on a Bruker Avance 400 at the Institute of Organic Chemistry (Heidelberg University). Proton broad band decoupling was applied for ^{13}C measurements. Deuterated solvents were used as purchased from Merck/Sigma-Aldrich or Deutero GmbH. Chemical shifts (reported in parts per million ppm) were referenced[1] to δ_H = 7.26 ppm ($CDCl_3$) for ^1H and δ_C = 77.16 ppm ($CDCl_3$) for ^{13}C and interpreted with MestReNova Version 14.1.2-25024. Apparent multiplicity is reported as s (singlet), d (doublet), dd (doublet of doublets), or m (multiplet).

Mass Spectrometry. Mass spectra and high-resolution mass spectra (MS/HRMS) were recorded at the Institute of Organic Chemistry (Heidelberg University). Matrix-assisted laser desorption/ionization (MALDI) and laser desorption/ionization (LDI) spectra were measured on a Bruker ApexQe hybrid 9.4 FT-ICR spectrometer or Bruker AutoFlex Speed time-of-flight spectrometer. DCTB (*trans*-2-[3-(4-*tert*-butylphenyl)2-methyl-2-propenylidene]malononitrile) was used as a matrix for the MALDI-MS experiments. Electron Ionization (EI) spectra were recorded on a JEOL AccuTOF GCx spectrometer.

Absorption Spectra. UV-Vis spectra were recorded on an Agilent Cary 60 UV-Vis spectrometer and measured in CH_2Cl_2 in the wavelength region of 230 to 800 nm under ambient conditions (rt: room temperature). The data obtained was interpreted with Spectra Manager from JASCO.

X-ray Crystallography. Single crystals were obtained by slow gas phase diffusion under the given conditions. The Bruker APEX-II Quazar diffractometer (radiation MoKα, λ = 0.71073 Å) with a CCD area detector and the STOE Stadivari instrument (radiation CuKα, λ=1.54178 Å) with a Pilatus CCD area detector (0.5° ω-scans) were used for data collection by the X-ray crystallography department at the Institute of Organic Chemistry (Heidelberg University). Structures were solved with the ShelXT[2] structure solution program and refined against F2 with a full-matrix least-squares algorithm with ShelXL.[3] Hydrogen atoms were treated with riding models. Graphic visualization and measurement of torsion angles were done with Mercury 2020.1.[4]

Infrared Spectroscopy. A JASCO FT/IR-4600 FTIR spectrometer was operated in ATR mode to record infrared spectra. The respective transmission spectra are baseline corrected, depicted in cm^{-1} and labeled according to the following abbreviations: s (strong), m (medium), w (weak).

Melting Point. The melting point was determined on a Büchi M-560 melting point apparatus in open capillaries. Decomp. refers to decomposition.

IUPAC Names and Formulas. IUPAC names and numbering of the synthesized compounds were generated using ACD/Labs 2021.

Computational Details.

DFT. Spin polarized Density Functional Theory calculations were performed as implemented in the FHI-aims code[5] to obtain the electronic properties of the precursor and **P-A, P-B, P-C** molecules. Their structural relaxation was done using the hybrid functional B3LYP(Becke, 3-parameter, Lee–Yang-Parr)[6]). All the calculations were carried up employing the Tkachenko-Scheffler treatment of the Van der Waals interactions.[7] Upon optimizing their atomic geometries, we calculated the frontier orbitals as well as the spin density maps before placing them at the Au(111) surface. The latter was modeled by a 10x12 slab consisting of 3 layers. All the atoms were thoroughly relaxed except the bottom Au layer. The atomic relaxation was finished when the remaining atomic forces and the total energy, were found below 10^{-5} eV and 10^{-2} eV/Å and respectively. A single gamma point was used for the integration of the Brillouin zone.

QM/MM. We performed QM/MM simulations employing the Fireball/AMBER software.[8] The metal surface representing the classical part was treated via the interface force-field. The quantum region including the molecules and one gold adatom, was described at the BLYP-DFT level with the Fireball code.[9] The calculated forces were managed via the Amber code and used to obtain the system dynamics.[10] First, we carried up a QM/MM geometry optimization followed by a thermalization from at 300K in order to stabilize the system. A Langevin thermostat was employed to simulate the canonical ensemble.[11] Then, a combination of the Steered Molecular Dynamics and the Umbrella Sampling Simulations was used to determine the free-energy profiles and the reactions mechanisms.[12-14] Note that the free-energy profile associated with each reaction was calculated using the Alan Grossfield´s implementation for the Weighted Histogram Analysis Method (WHAM).[15]

dI/dV simulations dI/dv maps were simulated using the PP-STM code[16] with a *CO* rigid tip. First, we calculated the eigenstates of the **P-A**, **P-B**, **P-C** molecules needed in the simulations, employing the hybrid functional B3LYP.[6] The dI/dV maps were acquired at different heights at the energy positions of the HOMO and LUMO orbitals respectively. The Lorentzian width parameter for broadening of the eigenstates was set to 0.005eV.

Quantum chemical calculations were performed using the Gaussian 16 program package.[17] Ground state geometry optimizations were performed by employing the B3LYP[6] functional, the 6-311G(d,p)[18] basis set and Grimme's D3 dispersion correction[19] with BJ-damping.[20] Thereby, ultra-tight convergence criteria of the respective computational method were used. Frequency calculations at the same level of theory were employed to verify the geometries as local minima possessing no imaginary frequencies. Nucleus independent chemical shifts (NICS) were calculated using the Gauge-Independent Atomic Orbital (GIAO)[21,22] approach, as implemented in Gaussian 16 at the GIAO-B3LYP(D3BJ)/6-311G(d,p) level of theory. Analysis of the results was done with the py.aroma software.[23] Calculation of HOMA values was accomplished with py.aroma software. Ring-current analysis was accomplished by using the Continuous Set of Gauge

Transformations (CSGT)[24] method at the CSGT-B3LYP(D3BJ)/6-311G(d,p) level of theory and visualized using the ACID program package of the Herges group.[25] Analysis of the π character of occupied orbitals was accomplished with the Multiwfn 3.8 software.[26]

Sample preparation and STM/nc-AFM experiments.

Experiments were performed in custom-designed ultra-high vacuum systems (base pressure below 5 x 10^{-10} mbar) hosting a commercial low-temperature microscope with STM/nc-AFM capabilities Createc GmbH. The Au(111) substrate (MaTeck GmbH) was cleaned by repeated cycles of Ar+ ion sputtering (E = 1 keV) and subsequent annealing to 740 K for 10 minutes. Molecular precursor 1 was thermally sublimed onto the clean Au(111) surface kept at RT (sublimation temperatures of 140°C). Unless otherwise noted, All STM images were taken in constant-current mode at a sample temperature of 5.0 K. Scanning parameters are specified in each figure caption. Non-contact AFM measurements were performed with Pt/Ir tip attached to a Qplus tuning fork[27] sensor from Createc. The tip was a posteriori functionalized by controlled adsorption of a single CO molecule at the tip apex from a previously CO-dosed surface.[28] The sensors were driven at their resonance frequency (30 kHz) with a constant amplitude of 50 pm. The frequency shift from the resonance of the sensor (with the attached CO-functionalized tip) was recorded in a constant-height mode (Nanonis SPM for Createc GmbH). STM and nc-AFM images were analyzed using WSxM software.[29]

Synthesis

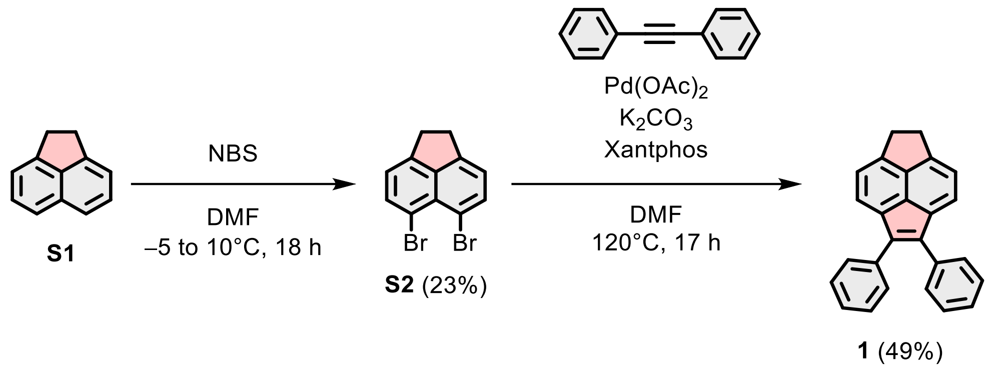

Scheme S1. Synthetic pathway towards compound **1**. NBS = *N*-bromosuccinimide, DMF = *N*,*N*-dimethylformamide, Xantphos = (9,9-dimethyl-9*H*-xanthene-4,5-diyl)bis(diphenylphosphane).

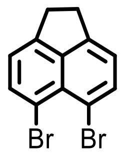

S2

5,6-Dibromo-1,2-dihydroacenaphthylene (S2). A three-neck flask was charged with DMF (500 mL) and acenaphthene (**S1**) (170 g, 1.10 mol) and cooled to –5 °C. Over a period of 4 h, NBS (500 g, 2.76 mmol) was added in four portions. After complete addition, the reaction was allowed to warm to 10 °C and stirred for 20 h. The formed precipitate was filtered off and the crude product was recrystallized from hot $CHCl_3$ (850 mL) to obtain **S2** (89.0 g, 286 mmol, 26%) as off-white crystals.

M.p.: 175 °C (lit. 172–173 °C).[30]

R_f = 0.60 (SiO_2, PE).

^{1}H NMR (400 MHz, $CDCl_3$, rt): δ = 7.80 (d, *J* = 7.4 Hz, 2H), 7.10 (d, *J* = 7.2 Hz, 2H), 3.31 (s, 4H) ppm.

^{13}C NMR (101 MHz, $CDCl_3$, rt): δ = 147.2, 142.2, 136.0, 128.0, 121.1, 114.6, 30.2 ppm.

EI-HRMS: *m/z* calcd. for $C_{12}H_8Br_2$: 309.8987 [M^+]; found: 309.8970.

Analytical data are consistent with the literature.[30]

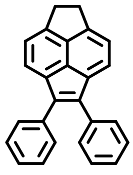

1

5,6-Diphenyl-1,2-dihydrocyclopenta[*fg*]acenaphthylene (1). A Schlenk flask was charged with dibromoacenaphthene **S2** (2.00 g, 6.41 mmol), tolane (1.37 g, 7.69 mmol), Xantphos (204 mg, 353 µmol), $Pd(OAc)_2$ (72.0 mg, 321 µmol) and K_2CO_3 (2.66 g, 19.2 mmol). The flask was evacuated and filled with nitrogen in three cycles. Degassed and dry DMF (20 mL) was added, and the resulting suspension was stirred at 120 °C for 16 h. The mixture was filtered through a plug of Celite, which was rinsed with CH_2Cl_2 (400 mL) and the solvents were removed under reduced pressure. The crude product was evaporated onto Celite and purified by flash column chromatography (SiO_2, PE/CH_2Cl_2 9:1) to give compound **1** (706 mg, 2.11 mmol, 33%) as an orange solid.

M.p.: 219–222 °C

R_f = 0.49 (SiO_2; petroleum ether/CH_2Cl_2 4:1)

^{1}H NMR (400 MHz, $CDCl_3$, rt): δ = 7.83 (d, J = 7.0 Hz, 2H), 7.52 (d, J = 8.2 Hz, 4H), 7.46 (d, J = 7.1 Hz, 2H), 7.37 (m, 4H), 7.30 (m, 2H), 3.56 (s, 4H) ppm.

(^{1}H NMR-data in agreement with the literature)[31]

^{13}C NMR (101 MHz, $CDCl_3$, rt) δ = 147.0, 137.8, 136.5, 135.9, 135.6, 130.2, 128.5, 127.0, 126.2, 121.0, 32.7 ppm. (1 signal coincident or not observed)

IR (FT-ATR) $\tilde{\nu}$ = 3022 (m), 2921 (w), 2853 (w), 2359 (w), 2337 (w), 1613 (w), 1598 (w), 1424 (m), 1051 (w), 1028 (w), 837 (s), 734 (m), 694 (s), 636 (m) cm^{-1}.

UV/Vis: (CH_2Cl_2, rt) λ_{max} (ε) = 249 (49400), 300 (5790), 333 (11000), 360 (12700), 427 (2160) nm (L mol^{-1} cm^{-1}).

EI-HRMS: *m/z* calcd. for $C_{26}H_{18}$: 330.1403 [M^+]; found: 330.1377.

NMR Data

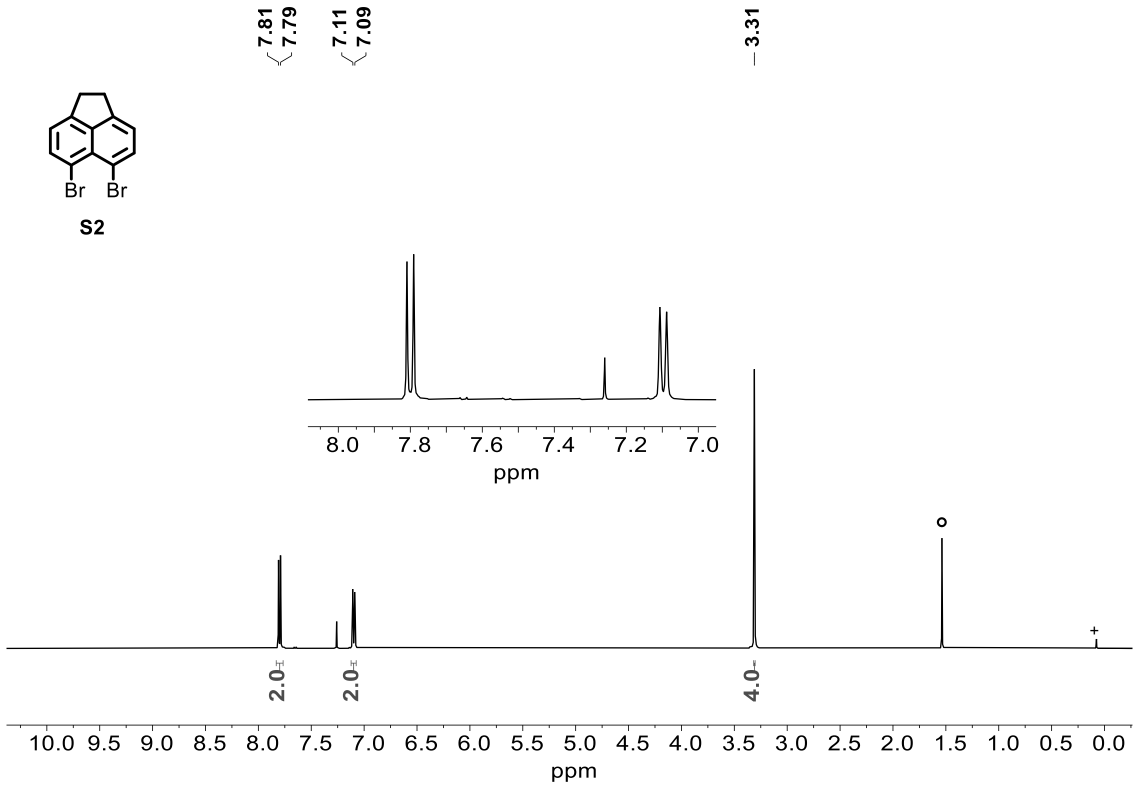

^{1}H NMR spectrum of **S2** (400 MHz, $CDCl_3$, rt); ° H_2O, $^{+}$ silicone grease.

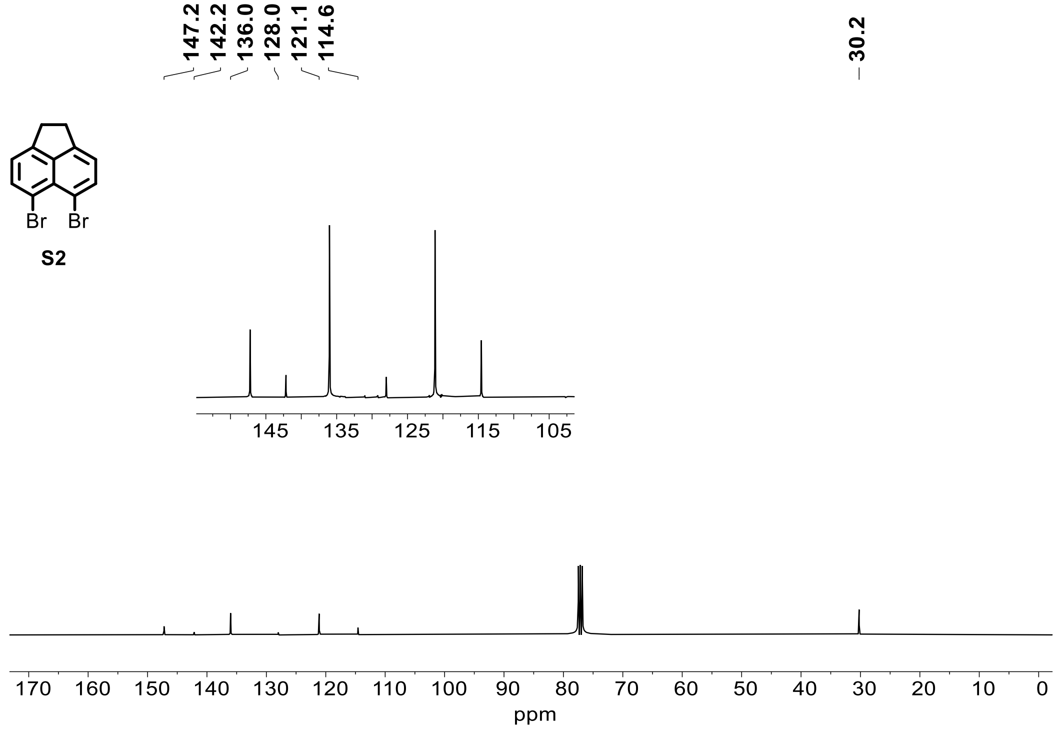

^{13}C NMR spectrum of **S2** (101 MHz, $CDCl_3$, rt).

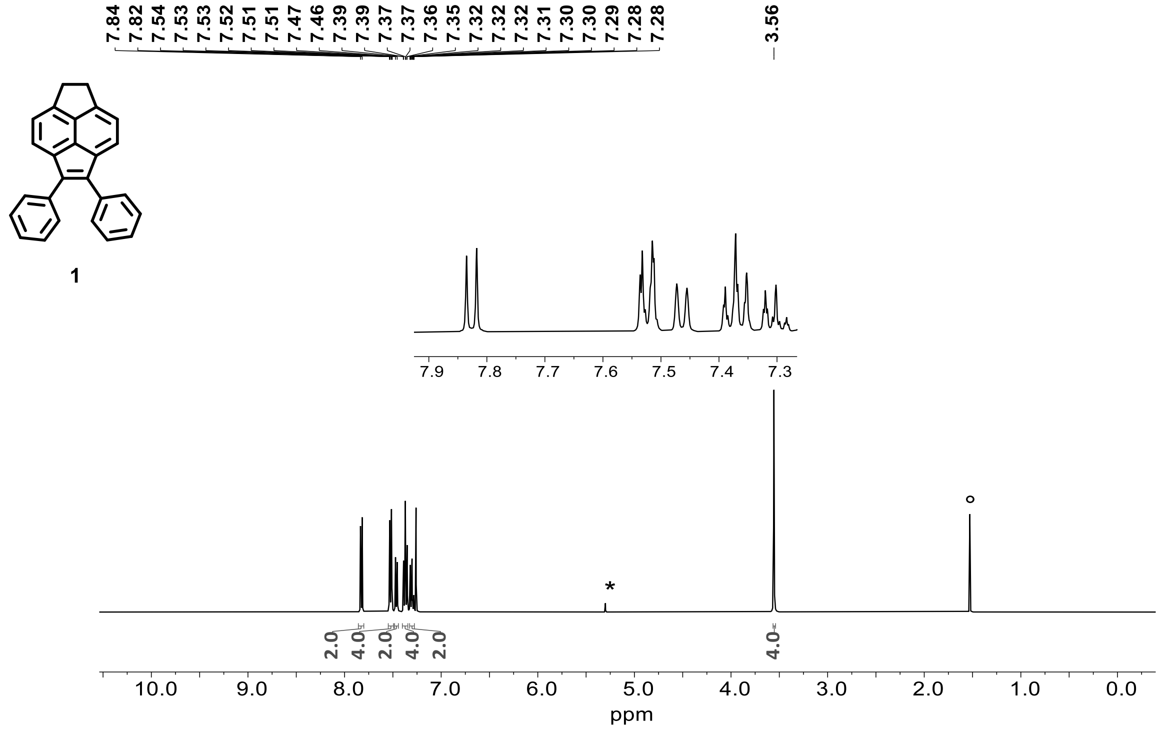

^{1}H NMR spectrum of compound **1** (400 MHz, $CDCl_3$, rt); °H_2O, *CH_2Cl_2.

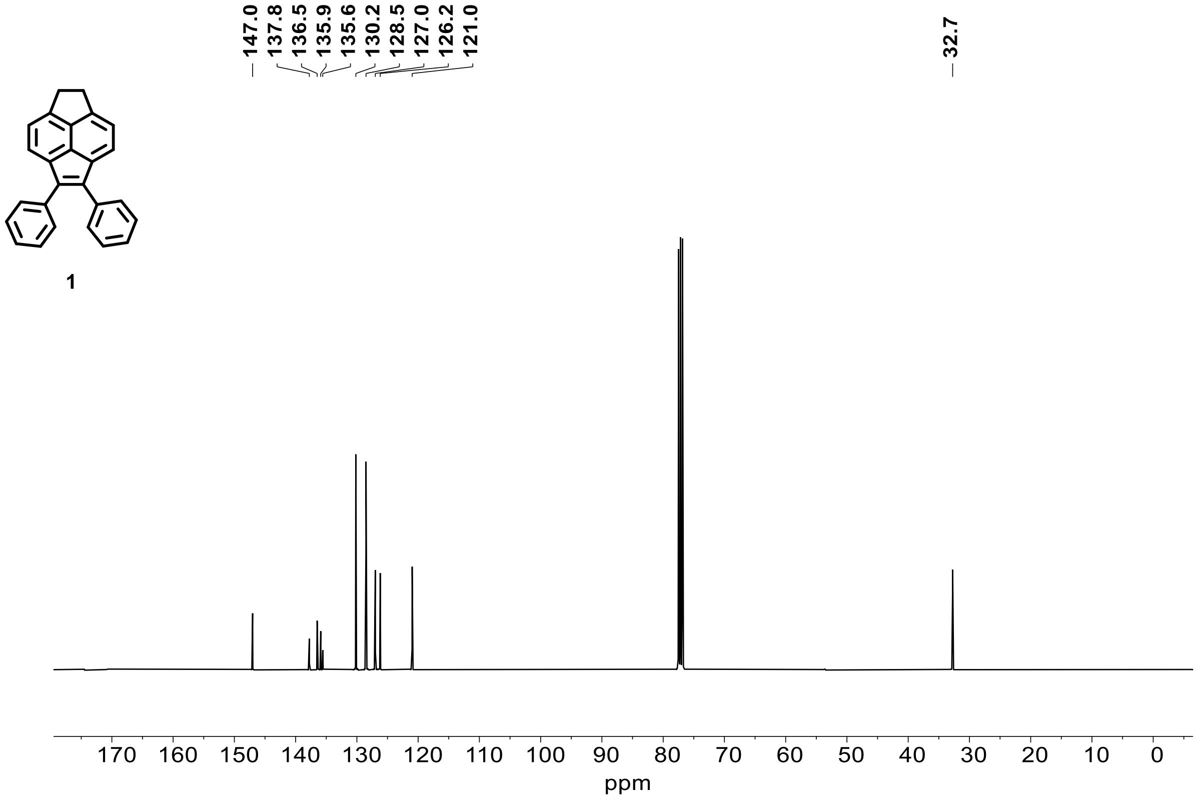

^{13}C NMR spectrum of compound **1** (101 MHz, $CDCl_3$, rt).

X-Ray Crystallographic Data

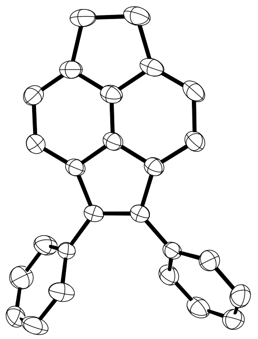

Single crystals of **1** suitable for X-ray crystallographic analysis were obtained by slow diffusion of pentanes into a solution of **1** in chloroform at room temperature.

Crystal data and structure refinement for compound **1**.

CCDC	2475868
Empirical Formula	$C_{26}H_{18}$
Formula Weight	330.40
Temperature	200(2) K
Wavelength	0.71073 Å
Crystal System	triclinic
Space Group	$P\bar{1}$
Z	2
Unit Cell Dimensions	a = 6.1568(6) Å α = 84.2329(17)°.
	b = 11.6618(12) Å β = 85.3439(17)°.
	c = 12.4435(13) Å γ = 77.5002(16)°.
Volume	866.22(15) Å^3
Density (Calculated)	1.27 gcm^{-3}
Absorption Coefficient	0.07 mm^{-1}
Crystal Shape / Color	column / orange
Crystal Size	0.413 x 0.049 x 0.048 mm^3
Theta Range for Data Collection	1.6 to 30.2 °.
Index Ranges	$-8 \leq h \leq 8$, $-16 \leq k \leq 15$, $-17 \leq l \leq 17$
Reflections Collected	17478
Reflections (Independent)	4752 (R(int) = 0.0479)
Goodness-of-fit on F^2	1.06
Final R Indices (I>2σ(I))	R1 = 0.057, wR2 = 0.132
Largest Diff. Peak and Hole	0.22 and –0.21 eÅ^{-3}

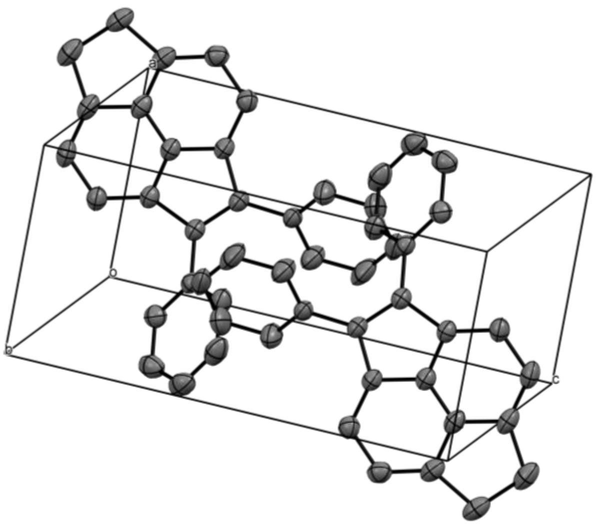

Unit cell of **1**. Ellipsoids at 50% probability level, hydrogen atoms and solvent molecules omitted for clarity.

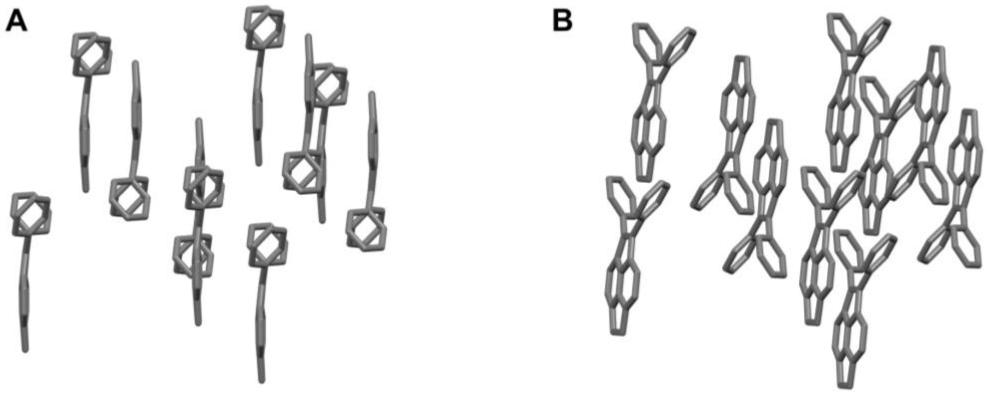

Representative cutout of organization of **1** in the solid state. **A.** Side view on stack of **1**. **B.** Twisted side view on stack of **1**.

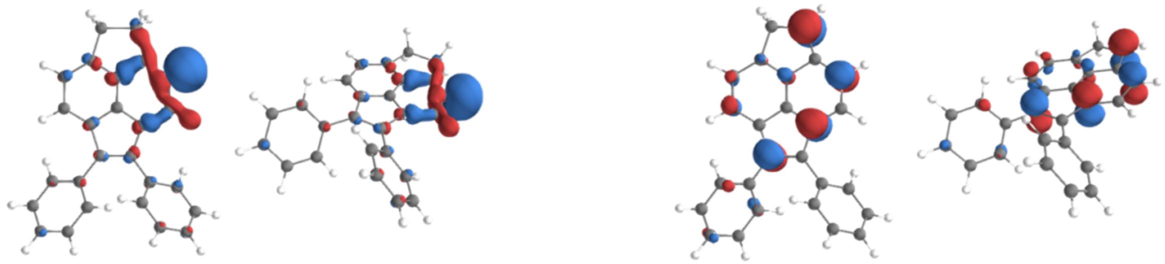

Figure S1. Singly occupied molecular orbitals (SOMOs) of the dehydrogenated precursor hosting either a σ-radical in the naphthalene subunit (left) or a π-radical at the saturated 5-membered ring (right) .

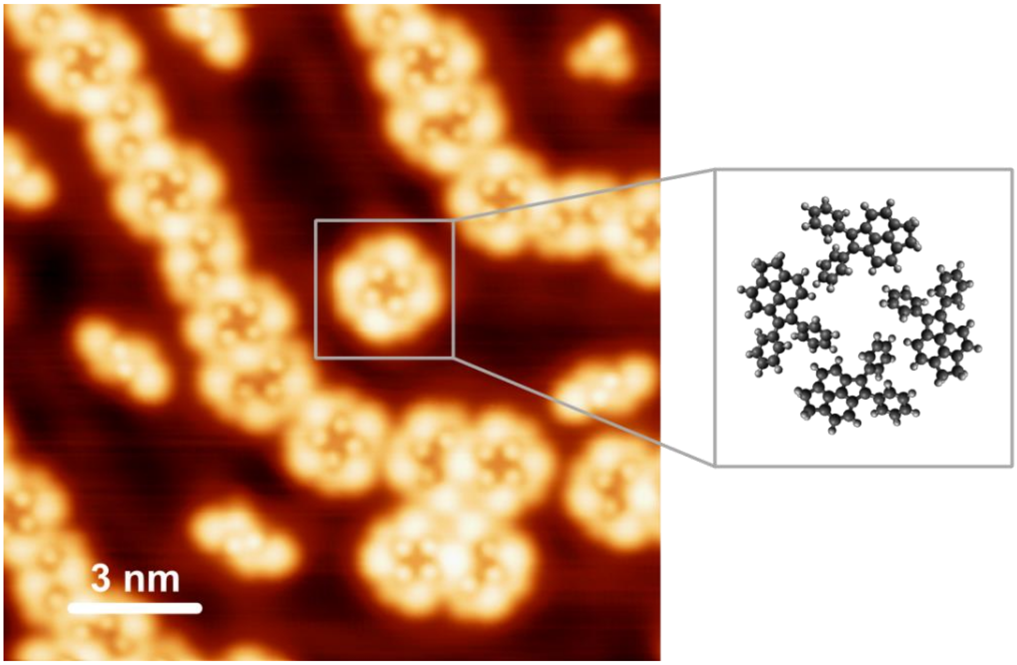

Figure S2. Self-assembly of precursor **1** on Au(111) after deposition with the substrate kept at RT. The gray square highlights a tetramer and a sketch of the self-assembly.

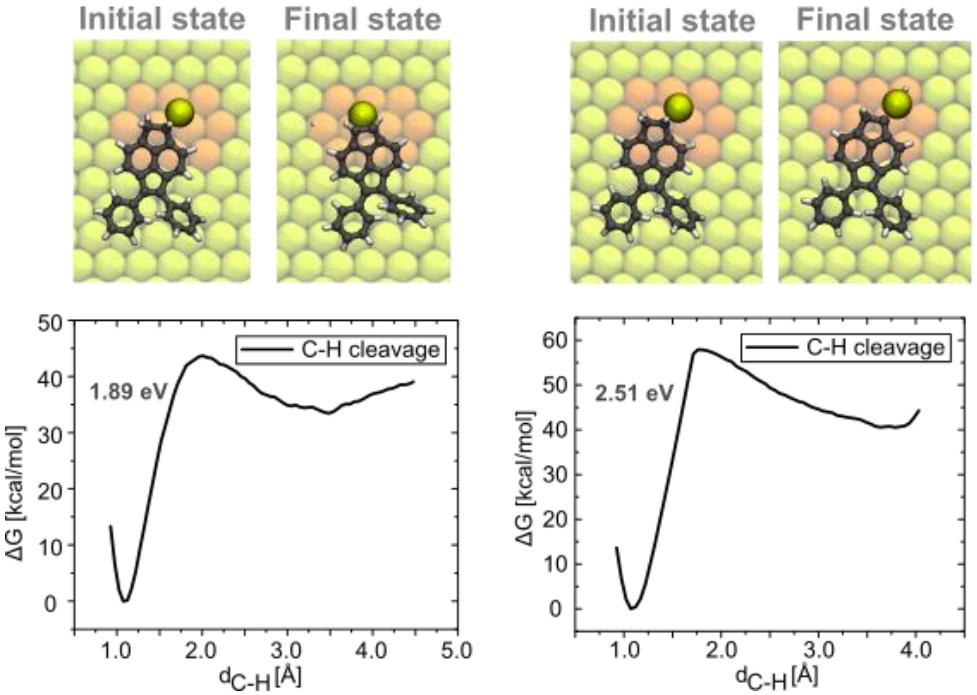

Figure S3. QM/MM free energy calculations of two possible competing reaction pathways upon Au-assisted C(sp^3)-H cleavage. C, H, Au adatom, Au surface atoms in MM region, and Au surface atoms in QM region are represented in black, white, golden yellow, light yellow, and orange balls, respectively.

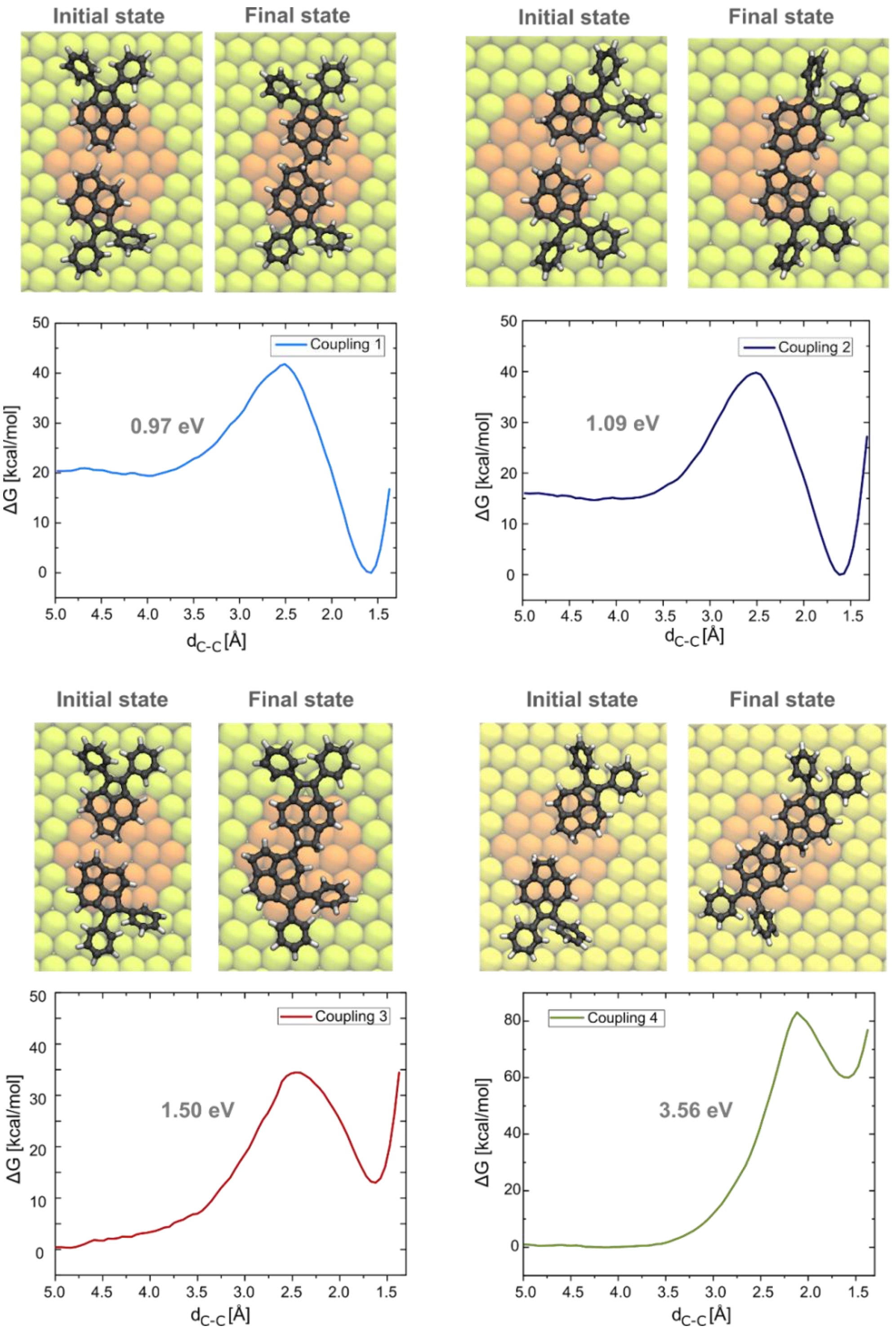

Figure S4. QM/MM free energy calculations of C-C coupling between π-radicals in two configurations (top panel), pi-radical and aromatic carbons (involved in the spin density, bottom left panel), and pi-radical and "inactivated" (closed shell) molecule (bottom right). C, H, Au surface atoms in MM region, and Au surface atoms in QM region are represented in black, white, yellow, and orange balls, respectively.

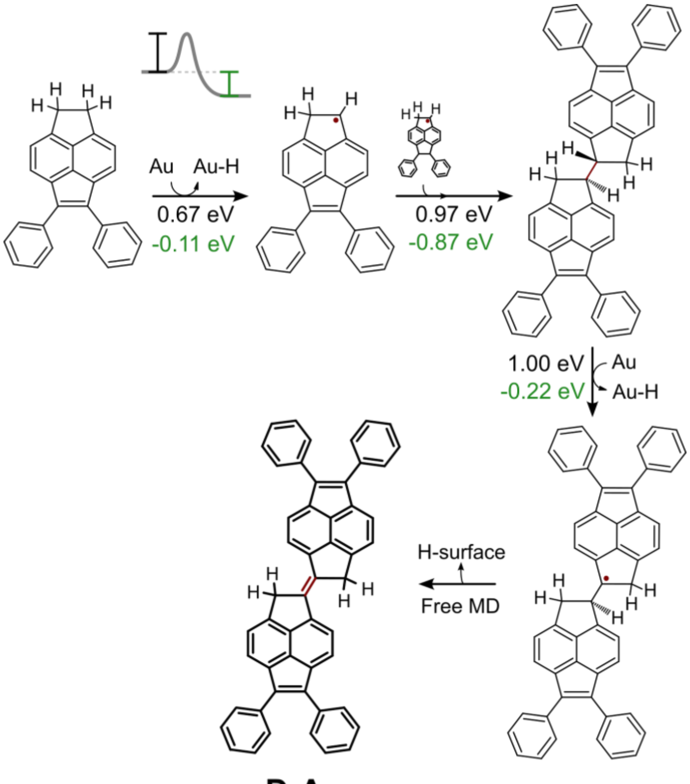

Figure S5. Proposed route towards the synthesis of **D-A**. The term H-surface refers to the dehydrogenation step with the help of the QM surface, where the H atom will attach to the QM surface. Each reaction step indicates the energy barrier (black) and energy difference between initial and final state (green).

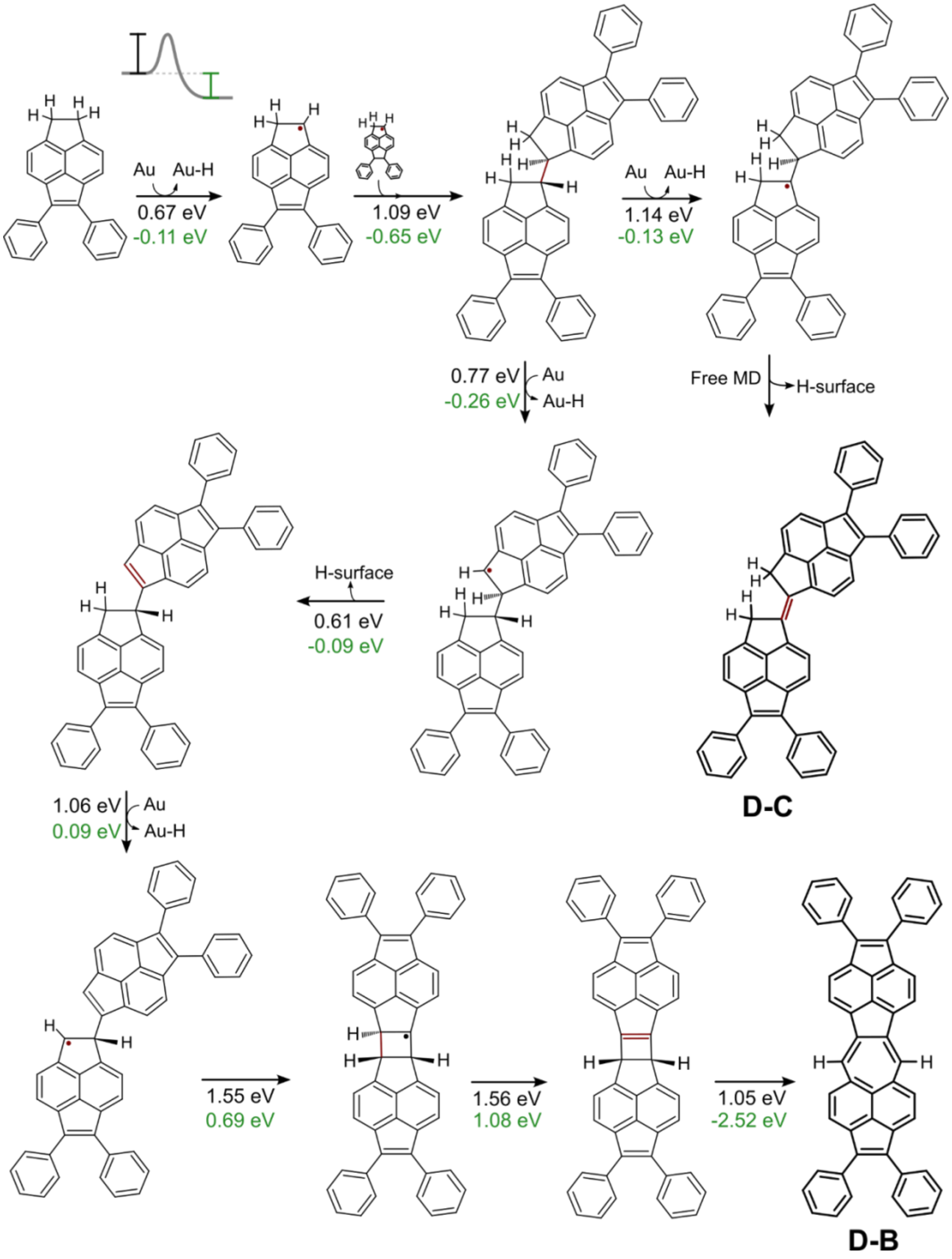

Figure S6. Proposed route towards the synthesis of **D-B** and **D-C**. The term H-surface refers to the dehydrogenation step with the help of the QM surface, where the H atom will attach to the QM surface. Each reaction step indicates the energy barrier (black) and energy difference between initial and final state (green).

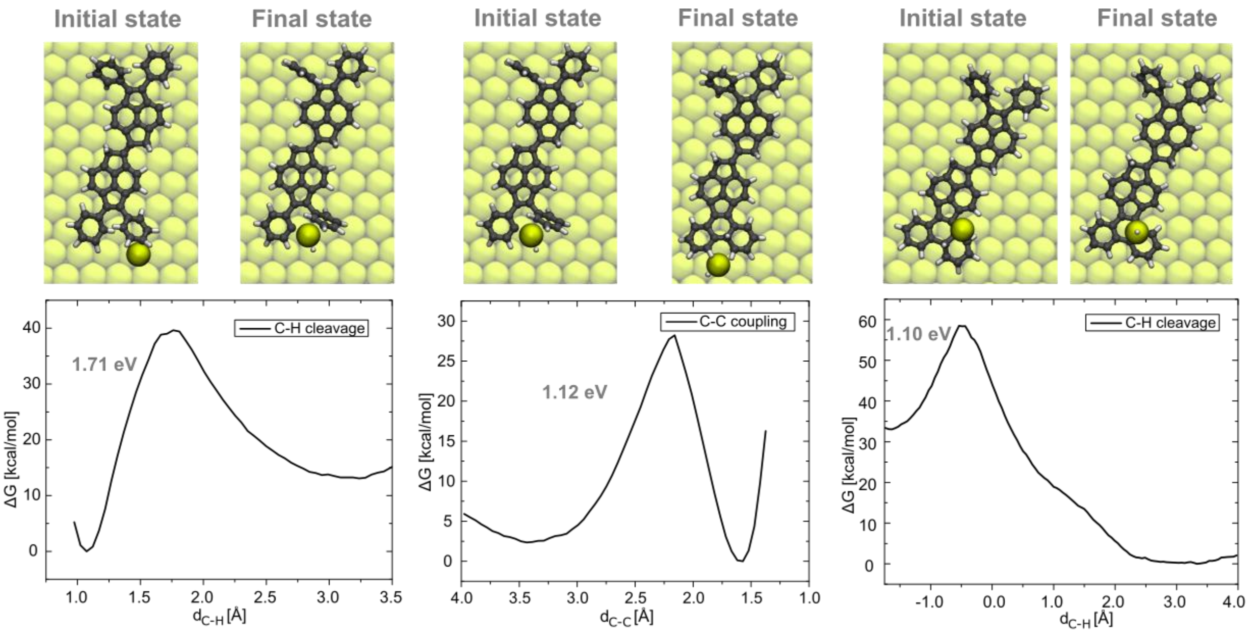

Figure S7. Cyclodehydrogenation process with the assistance of Au adatom, consisting of three subsequent steps: 1st step) represents the dehydrogenation of the phenyl group that is close to the surface assisted by an Au adatom; 2nd step shows the C-C coupling; 3rd step shows the last dehydrogenation reaction of the top H with the assistance of the Au adatom. C, H, Au adatoms, Au surface atoms in the MM region are represented in black, white, golden yellow, and light yellow balls, respectively.

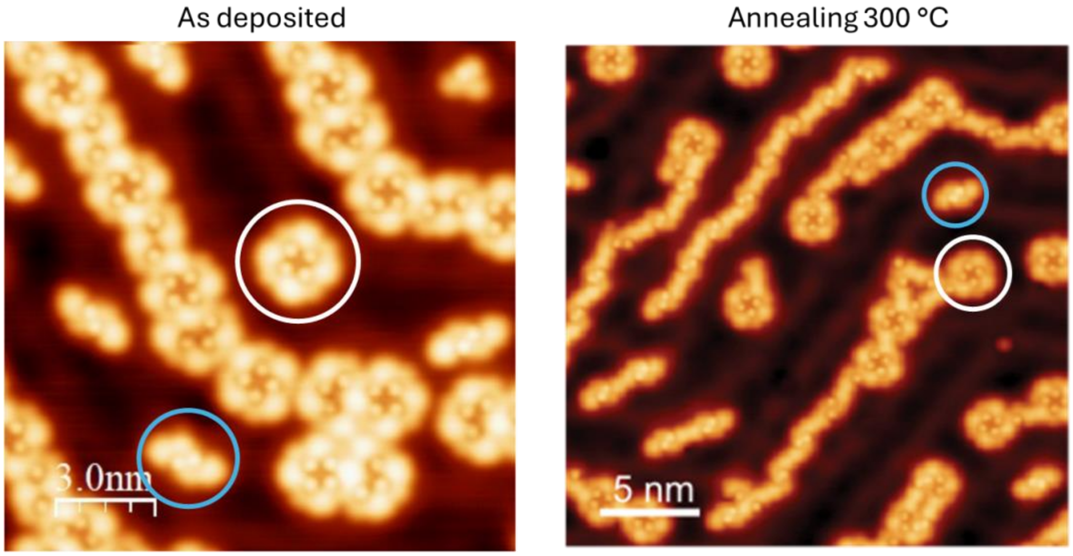

Figure S8. Comparison between as-deposited molecules and after thermal activation at 300°C. Some intact molecules are still present after annealing, assembling in dimers and tetramers as marked by blue and white circles, respectively.

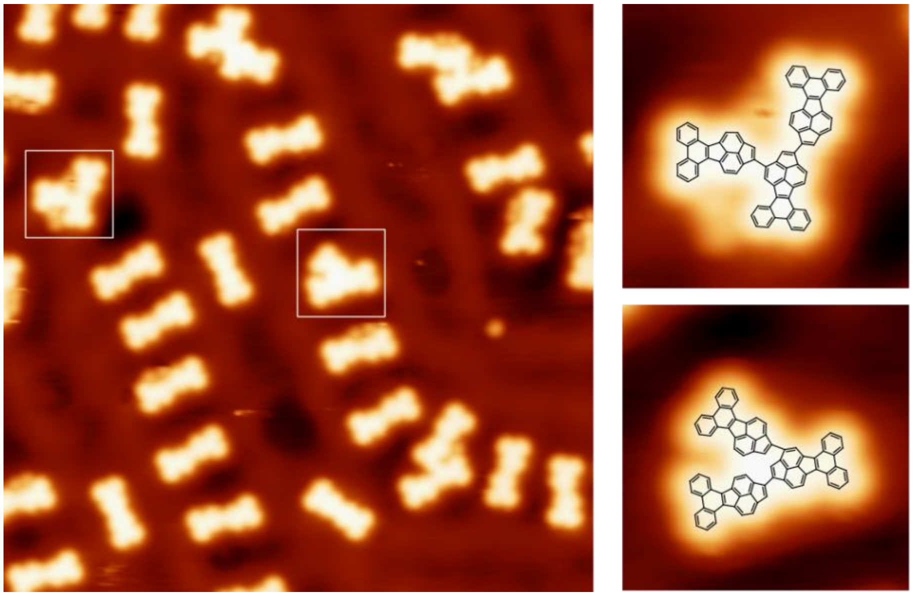

Figure S9. STM overview after annealing at 400°C (20x20 nm, left) and characterization of minority products resulting from the reaction between a pi-radical and an aromatic carbon (3.5x3.5 nm, right). V_b=100 mV, I_t = 30 pA for all images.

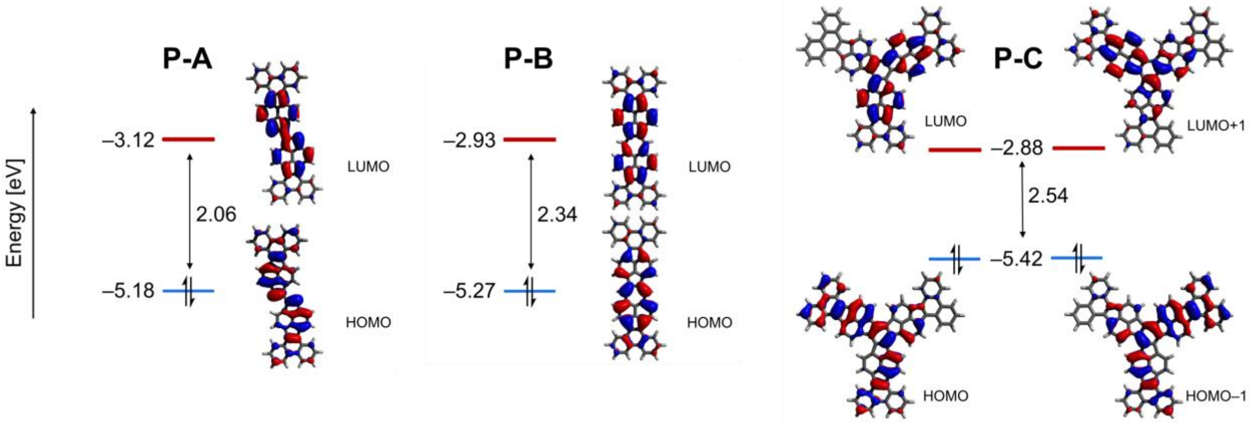

Figure S10. Electronic structure based on DFT calculations in the gas phase. Frontier molecular orbitals (FMOs) as calculated by DFT at the B3LYP(D3BJ)/6-311G(d,p) level of theory in the gas phase of **P-A**, **P-B** and **P-C**. Orbital energies are given in eV. Iso-surfaces plotted at 0.02 $Bohr^{-3/2}$.

Aromaticity Analysis

HOMA values

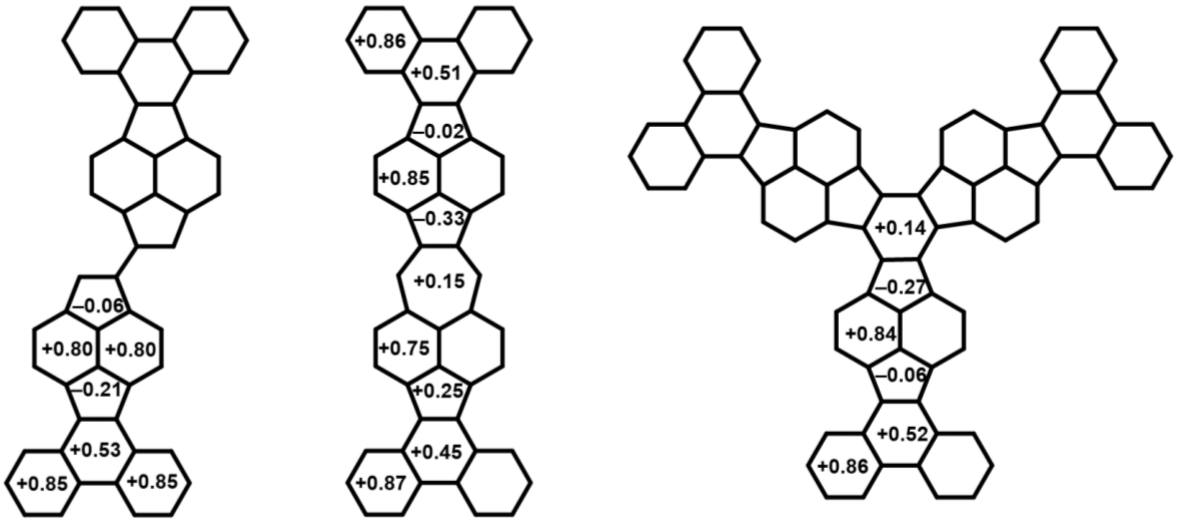

Figure S11. HOMA values of compounds **P-A** (left), **P-B** (middle), and **P-C** (right) based on the DFT optimized structures at the B3LYP(D3BJ)/6-311G(d,p) level of theory

NICS values

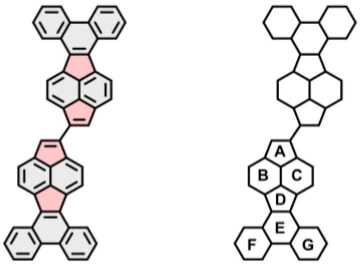

Table S1. NICS(1)$_{zz}$ values of **P-A** calculated at the B3LYP(D3BJ)/6-311G(d,p) level of theory.

	**A**	**B**	**C**	**D**	**E**	**F**	**G**
**P-A**	+33.0	–3.77	–0.64	+37.9	–13.0	–24.8	–23.6

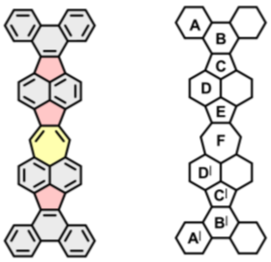

Table S2. NICS(1)$_{zz}$ values of **P-B** calculated at the B3LYP(D3BJ)/6-311G(d,p) level of theory.

	**A**	**B**	**C**	**D**	**E**	**F**
	–25.1	–14.3	+19.4	–12.7	+9.56	+2.86
**P-B**	**A**^{I}	**B**^{I}	**C**^{I}	**D**^{I}		
	–26.2	–14.0	–4.75	–19.3		

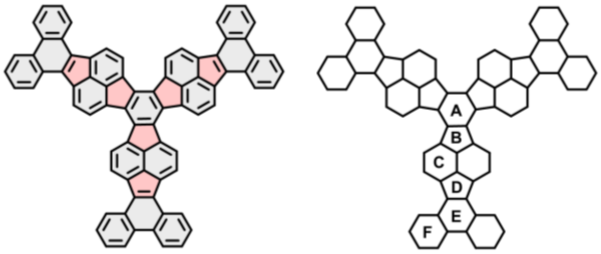

Table S3. NICS(1)$_{zz}$ values of **P-C** calculated at the B3LYP(D3BJ)/6-311G(d,p) level of theory.

	**A**	**B**	**C**	**D**	**E**	**F**
**P-C**	+12.3	+16.7	–10.6	+23.6	–14.1	–24.5

ACID plots

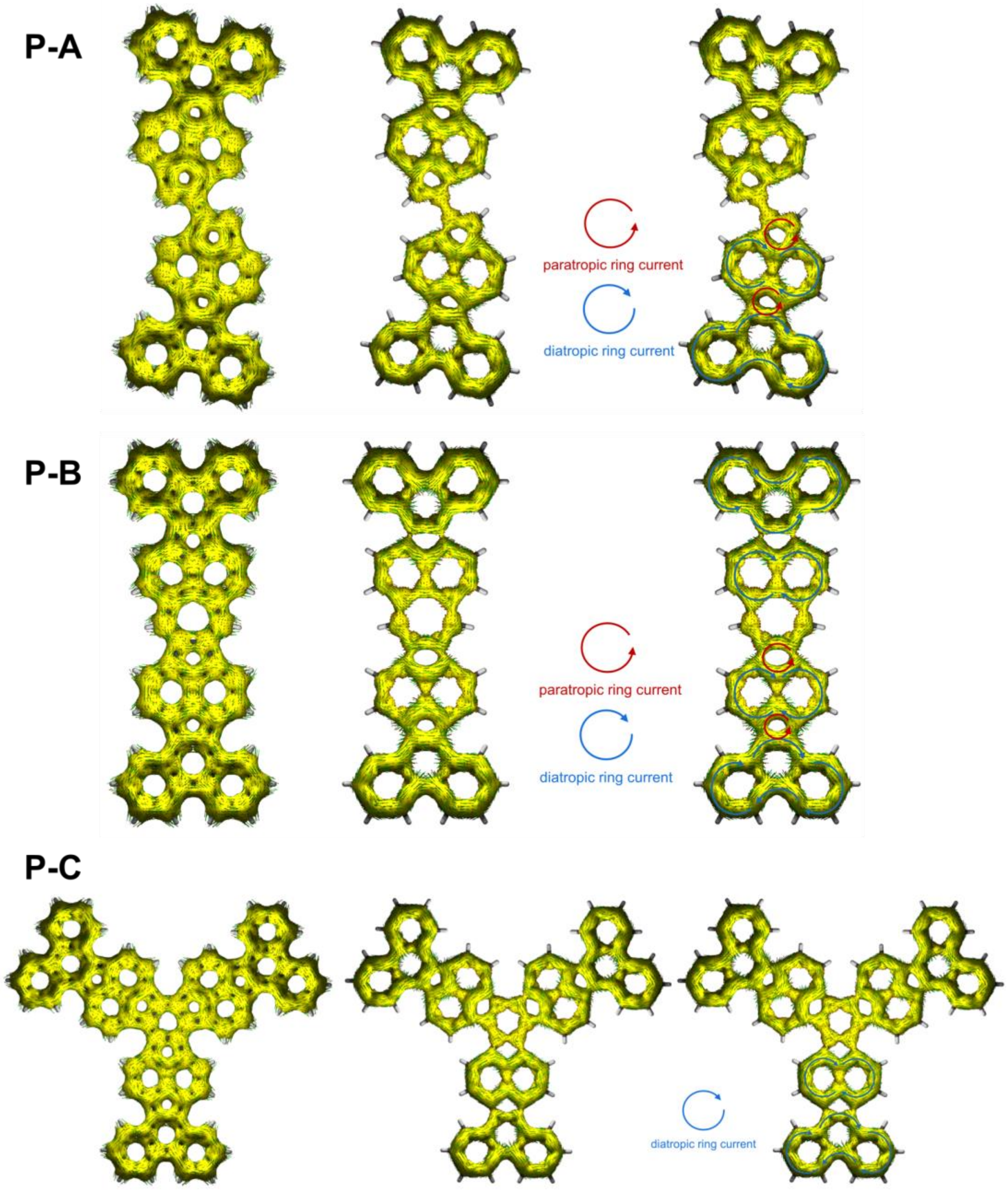

Figure S12. ACID plots of **P-A**, **P-B** and **P-C** computed at the B3LYP(D3BJ)/6-311G(d,p) level of theory. For the calculation all occupied orbital (left) or only the π orbitals (middle) are considered. π-Only ACID plot with the diatropic and paratropic are highlighted with blue and red arrows, respectively (right). The magnetic field is oriented orthogonally with respect to the paper plane.

References

1. G. R. Fulmer, A. J. M. Miller, N. H. Sherden, H. E. Gottlieb, A. Nudelman, B. M. Stoltz, J. E. Bercaw, K. I. Goldberg, *Organometallics* **2010**, *29*, 2176.

2. G. M. Sheldrick, *Acta Crystallogr., Sect. A: Found. Crystallogr.* **2008**, *64*, 112.

3. G. M. Sheldrick, *Acta Crystallogr., Sect. C: Cryst. Struct. Commun.* **2015**, *71*, 3.

4. C. F. Macrae, P. R. Edgington, P. McCabe, E. Pidcock, G. P. Shields, R. Taylor, M. Towler, J. van de Streek, *J. Appl. Crystallogr.* **2006**, *39*, 453.

5. V. Blum, *et al.*, *Comput. Phys. Commun.* **2009**, *180*, 2175.

6. a) P. J. Stephens, F. J. Devlin, C. F. Chabalowski, M. J. Frisch, *J. Phys. Chem.* **1994**, *98*, 11623; b) A. D. Becke, *J. Chem. Phys.* **1993**, *98*, 5648; c) C. Lee, W. Yang, R. G. Parr, *Phys. Rev. B* **1988**, *37*, 785; d) S. H. Vosko, L. Wilk, M. Nusair, *Can. J. Phys.* **1980**, *58*, 1200.

7. A. Tkatchenko, M. Scheffler, *Phys. Rev. Lett.* **2009**, *102*, 073005.

8. J. I. Mendieta-Moreno, R. C. Walker, J. P. Lewis, P. Gómez-Puertas, J. Mendieta, J. Ortega, *J. Chem. Theory Comput.* **2014**, *10*, 2185.

9. J. P. Lewis, P. Jelínek, J. Ortega, A. A. Demkov, D. G. Trabada, B. Haycock, H. Wang, G. Adams, J. K. Tomfohr, E. Abad, H. Wang, D. A. Drabold, *Phys. Status Solidi B* **2011**, *248*, 1989.

10. D. A. Pearlman, D. A. Case, J. W. Caldwell, W. S. Ross, T. E. Cheatham, S. DeBolt, D. Ferguson, G. Seibel, P. Kollman, *Comput. Phys. Commun.* **1995**, *91*, 1.

11. R. W. Pastor, B. R. Brooks, A. Szabo, *Mol. Phys.* **1988**, *65*, 1409.

12. D. A. Case, T. E. Cheatham III, T. Darden, H. Gohlke, R. Luo, K. M. Merz Jr., A. Onufriev, C. Simmerling, B. Wang, R. J. Woods, *J. Comput. Chem.* **2005**, *26*, 1668.

13. E. Darve, A. Pohorille, *J. Chem. Phys.* **2001**, *115*, 9169.

14. S. Izrailev, S. Stepaniants, B. Isralewitz, D. Kosztin, H. Lu, F. Molnar, W. Wriggers, K. Schulten, *Computational Molecular Dynamics: Challenges, Methods, Ideas* **1999**, 39.

15. A. Grossfield, WHAM: an implementation of the weighted histogram analysis method **2017**, version 2.1.0.

16. O. Krejčí, P. Hapala, M. Ondráček, P. Jelínek, *Phys. Rev. B* **2017**, *95*, 045407.

17. M. J. Frisch, G. W. Trucks, H. B. Schlegel, G. E. Scuseria, M. A. Robb, J. R. Cheeseman, G. Scalmani, V. Barone, G. A. Petersson, H. Nakatsuji, X. Li, M. Caricato, A. V. Marenich, J. Bloino, B. G. Janesko, R. Gomperts, B. Mennucci, H. P. Hratchian, J. V. Ortiz, A. F. Izmaylov, J. L. Sonnenberg, D. Williams, F. Ding, F. Lipparini, F. Egidi, J. Goings, B. Peng, A. Petrone, T. Henderson, D. Ranasinghe, V. G. Zakrzewski, J. Gao, N. Rega, G. Zheng, W. Liang, M. Hada, M. Ehara, K. Toyota, R. Fukuda, J. Hasegawa, M. Ishida, T. Nakajima, Y. Honda, O. Kitao, H. Nakai, T. Vreven, K. Throssell, J. A. Montgomery Jr., J. E. Peralta, F. Ogliaro, M. J. Bearpark, J. J. Heyd, E. N. Brothers, K. N. Kudin, V. N. Staroverov, T. A. Keith, R. Kobayashi, J. Normand, K. Raghavachari, A. P. Rendell, J. C. Burant, S. S. Iyengar, J. Tomasi, M. Cossi, J. M. Millam, M. Klene, C. Adamo, R. Cammi, J. W. Ochterski, R. L. Martin, K. Morokuma, O. Farkas, J. B. Foresman, D. J. Fox, *Gaussian 16 Rev. C.01* **2016**, Wallingford, CT.

18. R. Krishnan, J. S. Binkley, R. Seeger, J. A. Pople, *J. Chem. Phys.* **1980**, *72*, 650.
19. S. Grimme, J. Antony, S. Ehrlich, H. Krieg, *J. Chem. Phys.* **2010**, *132*, 154104.
20. S. Grimme, S. Ehrlich, L. Goerigk, *J. Comput. Chem.* **2011**, *32*, 1456.
21. J. R. Cheeseman, G. W. Trucks, T. A. Keith, M. J. Frisch, *J. Chem. Phys.* **1996**, *104*, 5497.
22. a) K. Wolinski, J. F. Hinton, P. Pulay, *J. Am. Chem. Soc.* **1990**, *112*, 8251; b) R. Ditchfield, *Mol. Phys.* **1974**, *27*, 789; c) R. McWeeny, *Phys. Rev.* **1962**, *126*, 1028; d) F. London, *J. Phys. Radium* **1937**, *8*, 397.
23. py.Aroma: An Intuitive Graphical User Interface for Diverse Aromaticity Analyses, *ChemRxiv* **2024**, doi:10.26434/chemrxiv-2024-mjmj8.
24. a) T. A. Keith, R. F. Bader, *Chem. Phys. Lett.* **1993**, *210*, 223; b) T. A. Keith, R. F. W. Bader, *Chem. Phys. Lett.* **1992**, *194*, 1.
25. a) D. Geuenich, K. Hess, F. Köhler, R. Herges, *Chem. Rev.* **2005**, *105*, 3758; b) R. Herges, D. Geuenich, *J. Phys. Chem. A* **2001**, *105*, 3214.
26. T. Lu, F. Chen, *J. Comput. Chem.* **2012**, *33*, 580.
27. F. J. Giessibl, *Rev. Sci. Instrum.* **2019**, *90*, 011101.
28. L. Gross, F. Mohn, N. Moll, P. Liljeroth, G. Meyer, *Science* **2009**, *325*, 1110.
29. I. Horcas, R. Fernández, J. M. Gómez-Rodríguez, J. Colchero, J. Gómez-Herrero, A. M. Baro, *Rev. Sci. Instrum.* **2007**, *78*, 013705.
30. J. Bergner, C. Walla, F. Rominger, A. Dreuw, M. Kivala, *Chem. Eur. J.* **2022**, *28*, e202201554.
31. W. An, G. Li, J. Ma, Y. Tian, F. Xu, *Synlett* **2014**, *25*, 1585.
